# Body size, shape and ecology in tetrapods

**DOI:** 10.1038/s41467-022-32028-2

**Published:** 2022-07-27

**Authors:** Alice E. Maher, Gustavo Burin, Philip G. Cox, Thomas W. Maddox, Susannah C. R. Maidment, Natalie Cooper, Emma R. Schachner, Karl T. Bates

**Affiliations:** 1 Department of Musculoskeletal & Ageing Science, Institute of Life Course & Medical Sciences, University of Liverpool, William Henry Duncan Building, 6 West Derby Street, Liverpool, L7 8TX UK; 2 Natural History Museum, London, Cromwell Road, London, SW7 5BD UK; 3 Department of Archaeology and Hull York Medical School, University of York, PalaeoHub, Wentworth Way, Heslington, York, YO10 5DD UK; 4 School of Veterinary Science, Institute of Infection, Veterinary and Ecological Sciences, University of Liverpool, Small Animal Teaching Hospital, Leahurst Campus, Chester High Road, Neston, CH64 7TE UK; 5 Department of Earth Sciences, Natural History Museum, London, Cromwell Road, London, SW7 5BD UK; 6 Department of Cell Biology & Anatomy, School of Medicine, Louisiana State University Health Sciences Center, New Orleans, LA USA

**Keywords:** Palaeontology, Biomechanics

## Abstract

Body size and shape play fundamental roles in organismal function and it is expected that animals may possess body proportions that are well-suited to their ecological niche. Tetrapods exhibit a diverse array of body shapes, but to date this diversity in body proportions and its relationship to ecology have not been systematically quantified. Using whole-body skeletal models of 410 extinct and extant tetrapods, we show that allometric relationships vary across individual body segments thereby yielding changes in overall body shape as size increases. However, we also find statistical support for quadratic relationships indicative of differential scaling in small-medium versus large animals. Comparisons of locomotor and dietary groups highlight key differences in body proportions that may mechanistically underlie occupation of major ecological niches. Our results emphasise the pivotal role of body proportions in the broad-scale ecological diversity of tetrapods.

## Abstract (web summary)

Here, the authors examine how body size, shape, and segment proportions correspond to ecology in models of 410 tetrapods. They find variable allometric relationships, differential scaling in small and large animals, and body proportions as a potential niche occupation mechanism.

## Introduction

Body size and shape play a universal and fundamental role in the mechanical and physiological function of all organisms^1–3^. At the most basic level, the motion of terrestrial vertebrates is constrained by Newtonian mechanics; that is, acceleration is a function of force and mass. Body proportions describe the magnitude and distribution of mass within the moving body and the lengths of levers responsible for generating that movement. Body shape also plays a determinant role at multiple physiological levels; for example, in describing the space available for accommodating major organ systems^4,5^, and body surface area for heat exchange^6,7^. Because different environments and behaviours place different demands on the functional mechanics and physiologies of organisms, it is expected that body proportions should vary across animals occupying different ecological niches^8–14^. However, modification of body size and shape by ecological pressures may also be constrained by other factors, notably phylogenetic history and the ecological trajectory of evolutionary change^3,15,16^.

Given the universal potential for natural selection to act upon body shape, it is not surprising that many studies have sought to investigate associations between body proportions and ecological niche occupation^8–14^. While these studies have regularly identified important trends in the evolution of body proportions^8–14^, they have tended to focus on individual taxonomic or ecological groups, or on an individual aspect of body shape. However, to our knowledge, no study has systematically investigated allometric patterns or ecological differences in whole-body proportions across a highly diverse sample of extinct and extant tetrapods.

**Fig. 1:**
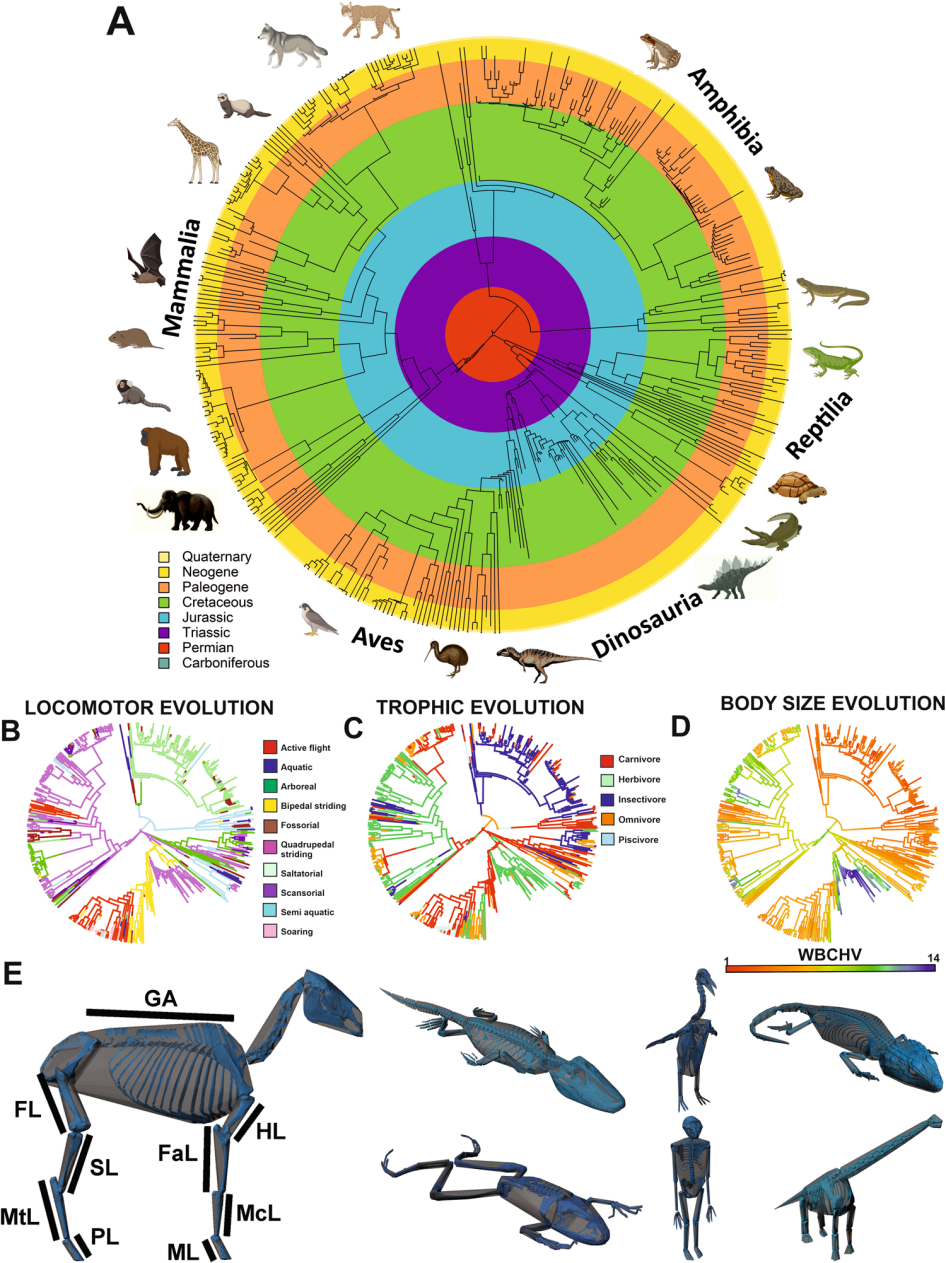
Body size, shape and ecology in tetrapods. To investigate the evolution of body shape and ecology in tetrapods we assembled a data set of **A** 410 extinct and extant terrestrial vertebrates from across Tetrapoda that captured major evolution changes in **B** locomotor and **C** trophic ecology and **D** body size. **E** From 3D digital skeletal models of these taxa we extracted a range of linear and volumetric measures and used them to derive measures of body size and shape using phylogenetic comparative approaches. Linear measurements included gleno-acetabular distance (GA), femur length (FL), shank segment length (SL), metatarsal segment length (MtL), pes segment length (PL), humerus length (HL), forearm segment length (FaL), metacarpal segment length (McL) and manus segment length (ML). WBCHV, whole-body convex hull volume. Animal images created with BioRender.com.

In this work we present a systematic statistical analysis of whole-body proportions across a broad sample of tetrapods using a dataset of 410 digital skeletons (Fig. 1). To address a series of hypotheses that examine the complex interaction among body size, shape and locomotor and trophic ecology, we extract not only linear measurements of body segment size from our 3D skeletal models, but also use mathematical shape-fitting to generate 3D volumetric representations of body proportions^17,18^ (Fig. 1). This allows us to examine changes in body segments whose overall size and shapes are poorly captured by linear measurements, and also provides a whole organism measure of body size against which to assess allometric changes in proportions. We show that allometric relationships vary across individual body segments thereby yielding changes in overall body shape as size increases, but also find statistical support for quadratic relationships indicative of differential scaling in small-medium versus large animals. Our results provide insights into how tetrapod body construction has been shaped as a multi-element or modular system in relation to locomotor and trophic ecology.

## Results and discussion

### (Hypothesis 1) Body shape is maintained (scales isometrically) across the full range of body sizes exhibited by terrestrial tetrapods

In the 20^th^ Century a series of landmark studies attempted to assess if major groups (e.g. mammals), or sub-groups (e.g. ungulates), exhibit consistent proportional changes in limb segment dimensions across large body size ranges^19–22^; in other words, do allometric constraints on animal mechanics and physiology^1–7^ impose a universal scaling pattern on body proportions? While previous studies have focused on allometric patterns in specific body segments within tetrapod sub-groups, we provide an examination of this hypothesis by analysing scaling patterns from all major body segments simultaneously (thereby examining whole-body shape change) in our broad sample of tetrapods (Fig. 1 and Supplementary Fig. 1), which includes some of the smallest (e.g., *Sorex monticolos*, *Selasophorus sasin*, *Takydromus sexlineatus*) with approximate masses of 0.005 kg, and the largest taxa (*Loxodonta africana*, *Tyrannosaurus rex*, *Dreadnoughtus schrani*), with estimated masses of up to ~40,000 kg^18,23^.

**Fig. 2:**
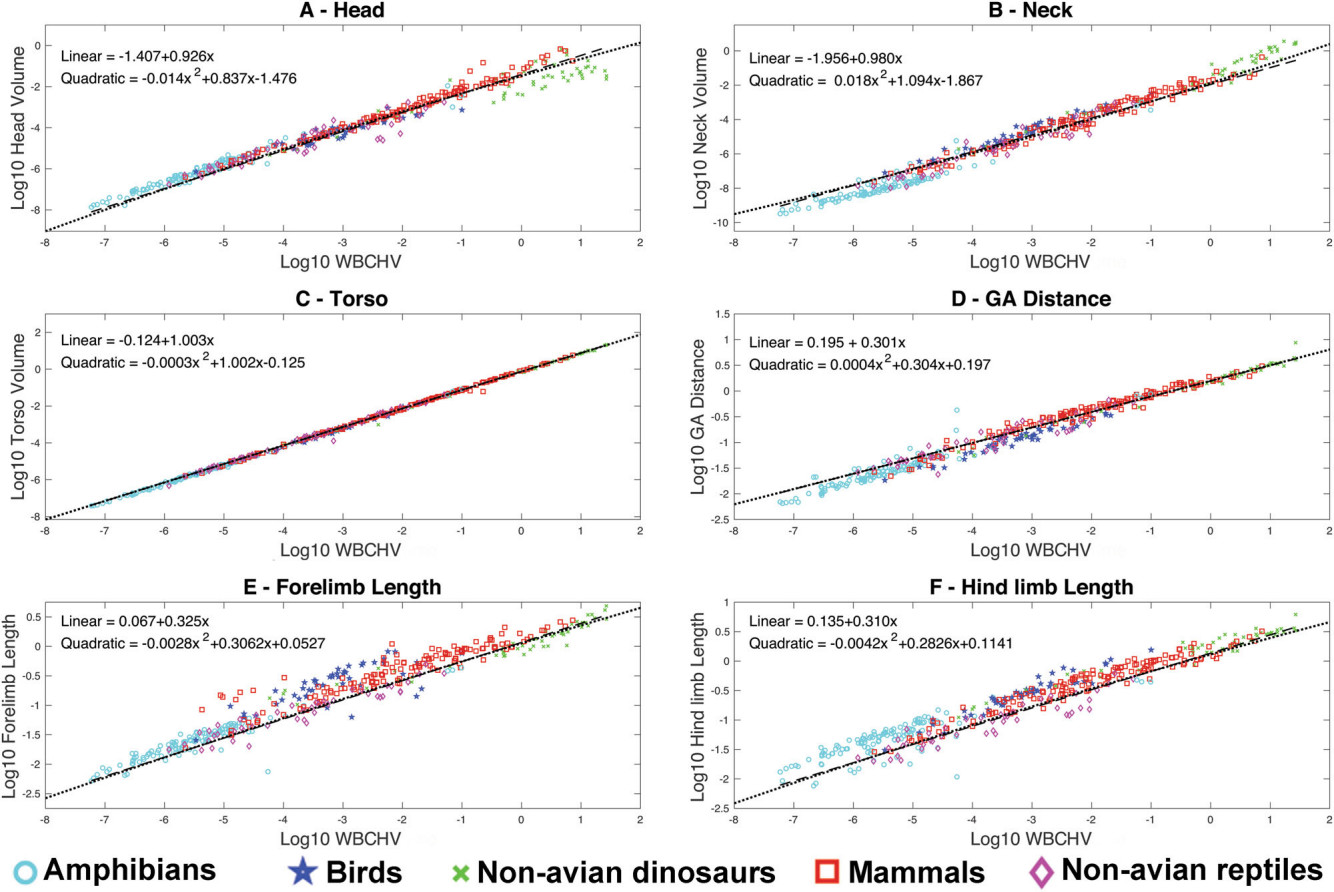
Scaling of major body segments in tetrapods. Scaling relationships between major body segment size and overall body size (total whole-body skeletal convex hull volume, WBCHV) in 410 terrestrial tetrapods using phylogenetically-informed linear (thick dashed lines) and quadratic (thin dotted lines) fits (Hypotheses 1-2). The **A** head, **B** neck and **C** torso are represented by volumes, while **D** gleno-acetabular (GA) distance, **E** total forelimb and **F** total hind limb size is represented by lengths. Isometry in **A**–**C** would therefore be a slope of 1, and in **D**–**F** a slope of 0.33. Full details of the regression model information can be found in Supplementary Data 1–14, including additional comparisons of scaling in individual limb segment lengths (Supplementary Fig. 1) and volumes. Taxa have been colour-coded by taxonomic order for display purposes. Source data are provided as a Source Data file.

Linear allometric relationships for all body segments are statistically significant (Fig. 2, Supplementary Data 1–2, Supplementary Fig. 1), with a relatively strong phylogenetic signal (λ values between 0.819-0.938). Phylogenetically generalised least squares (PGLS) regression^24^ slopes for the femur, humerus, forearm, metacarpal, overall forelimb length, torso and neck volume are indistinguishable from isometry, providing support for broad geometric similarity in these segments across the full body size range seen in tetrapods (Fig. 2, Supplementary Data 1–2, Supplementary Fig. 1). Negative allometry is recovered for the shank, metatarsal, pes, manus, gleno-acetabular (GA) distance and overall hind limb length, and skull volume (Fig. 2, Supplementary Data 1–2, Supplementary Fig. 1). The magnitude of negative allometry increases from proximal to distal segments within the hind limbs of tetrapods, consistent with more ‘graviportal’ limb proportions in large versus small animals^25,26^. However, we find little support for this proximal to distal trend in the forelimb. This suggests that, across tetrapods as a whole, size-based constraints on forelimb construction are matched by selective pressures associated with highly disparate functional mechanics in different locomotor ecologies (e.g., flight, fossoriality, arboreality; see below). The metatarsal and pes segments show the strongest negative allometry, but confidence intervals do not come close to elastic similarity^20,27^ (e.g., metatarsal segment lower 95% CI = 0.278). Isometric scaling of torso volume but negative allometry in GA distance suggests a change in torso shape characterised by mediolateral expansion of the ribcage and pelvis as body size increases in tetrapods. Therefore, contra to Hypothesis 1, tetrapods change body shape as size increases: relative head size decreases, the torso becomes wider but anteroposteriorly shorter, and the hind limb becomes strongly graviportal, but the forelimb only weakly so (Fig. 2 and Supplementary Fig. 1).

### (Hypothesis 2) Body shape scales non-linearly across the full range of body sizes exhibited by terrestrial tetrapods

Studies of specific body segments in certain tetrapod sub-groups have proposed size thresholds in animal function, above which aspects of anatomy and biomechanics, such as effective limb mechanical advantage and maximum performance (e.g., running speed), differ in smaller versus larger animals^20–23,27–32^. Attempts to correlate size-thresholds in mechanics and physiology to causative non-linear changes in morphology have recovered mixed results^20,23,27^, but to date these studies have been restricted to specific taxonomic orders and individual body segments. We find statistically significant (*p* < 0.05) second-degree coefficients for phylogenetically-informed quadratic models fit through all our linear and volumetric body segment measurements (Fig. 2; Supplementary Data 3–4, Supplementary Fig. 1). However, in most cases, lower sample size corrected Akaike Information Criterion (AICc) values suggest that phylogenetically-informed linear models are slightly better supported than quadratic models across tetrapods as a whole (Fig. 2; Supplementary Data 3–4, Supplementary Fig. 1). In most cases, AICc values are very similar, and values for shank length, metatarsal length, skull volume and neck volume are lower for the phylogenetically-informed quadratic models, providing statistical support for non-linear scaling in these body segments across tetrapods as a whole. We therefore find mixed support for Hypothesis 2 across tetrapods.

Taxa with upright striding gaits define the extremes of body size in tetrapods, and their locomotor systems are under the strongest or narrowest selection pressure in terms of first-order Newtonian mechanics. That is, locomotion in these groups is mostly dedicated to anti-gravity support on the ground, while other groups are subject to additional selective pressures (e.g. flight, swimming, climbing, burrowing). Linear and quadratic models fitted through data for bipedal and quadrupedal striding categories are again predominantly statistically significant (*p* < 0.05; Supplementary Data 5–8) with very similar AICc values in most cases. In bipedal striding tetrapods, phylogenetically-informed linear models are statistically better supported than quadratic models for hind limb segments and overall hind limb length, while phylogenetically-informed quadratic models better describe scaling trends in most forelimb segments and the forelimb overall (Supplementary Data 5 and 6). These trends may reflect highly varied ecological function of the forelimb across bipedal striding taxa. Smaller bipedal taxa are predominantly extant flightless birds which have retained relatively long forelimb segments despite flight loss, while larger bipedal taxa are generally non-avian theropod dinosaurs with short forelimbs, which may have been actively used in prey capture rather than body support during locomotion.

In upright quadrupedal striding tetrapods, which include the largest mammals and dinosaurs, we find strong statistical evidence that quadratic models best describe scaling of the gross locomotor system (Supplementary Data 7 and 8), thereby providing support for Hypothesis 2 within this locomotor group. Lengths and skeletal volumes for the hind limb, forelimb and all individual limb segments with the exception of the femur (length and volume) and humerus (length) are better described by phylogenetically-informed quadratic rather than linear models (Supplementary Data 7 and 8). This therefore provides initial evidence for near-ubiquitous differential size-based scaling in gross locomotor anatomy in quadrupedal tetrapods. However, these relationships are highly complex, and it is possible that the recovered quadratic relationships are influenced by non-selective allometric constraints or inherent methodological assumptions, such as the assumed evolutionary model (Brownian Motion) and/or data transformations. Caution in proposing selective factors, such as locomotor biomechanics, as the sole causative factor of the recovered non-linearity is therefore warranted. While quadratics fits remain more strongly supported for quadrupedal striding taxa using ordinary least squares regression (i.e. no evolutionary model; Data S7–8), an extended statistical approach that allows explicit evaluation of data treatment and particularly assumptions of phenotypic evolution in quadratic models is required to directly and more quantitatively establish the biological foundation of the recovered patterns.

To examine the nature of this potential non-linearity in body segment allometry outside of more complex quadratic fits, we compared the linear slopes of a series of size thresholds (or bins) within the full data set and within upright quadrupedal striding taxa (Supplementary Data 9–14; Supplementary Fig. 2). For the more distal limb segments the qualitative difference between slopes remains similar regardless of the specific body size threshold (approximately 25 kg, 100 kg or 500 kg) chosen to split the data into ‘smaller’ vs ‘larger’ animals; that is, in all these limb segments (metatarsal, pes, metacarpal and manus) the larger size group always displays stronger negative allometry than the corresponding smaller size group (Supplementary Data 9–14; Supplementary Fig. 2). The next most proximal segments in the forelimb (i.e., forearm segment) and hind limb (i.e., thigh and shank segments) show more negative allometry in the “larger” size bin when animals are split at thresholds of approximately 25 kg or 100 kg body mass. However, when split at a threshold of approximately 500 kg, a reversal of the scaling pattern is found, with animals larger than 500 kg scaling with less negative allometry than animals smaller than 500 kg. In the humerus the relative reversal of slopes occurs at the lower mass threshold of 100 kg (Supplementary Data 9–14; Supplementary Fig. 2). This reversal is such that animals over approximately 500 kg show positive allometry in these segments, particularly in the humerus (Supplementary Data 9–14; Supplementary Fig. 2).

**Fig. 3:**
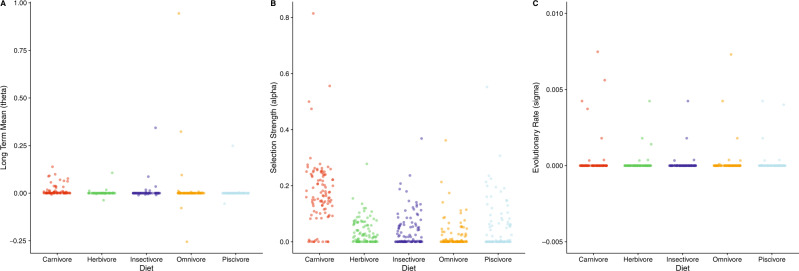
Models of body size evolution in tetrapods. Parameter estimates from the best fitting model of body size evolution under different evolutionary regimes defined by trophic ecology (80 OUMA and 20 OUMVA), for 100 sampled simulated evolutionary histories. **A** Long-term mean (θ), **B** selection strength (α), and **C** evolutionary rate (σ^2^) (Hypothesis 3). Each point corresponds to the parameter estimate for one of the sampled simulated evolutionary histories. Source data are provided as a Source Data file.

While we emphasise that further work is required is to separate out the contribution of methodological assumptions and non-selective mechanisms to non-linear scaling of body proportions (Fig. 3 and Supplementary Data 9–14), it is plausible that the non-linear relationships recovered here may also reflect selective factors thought to act on vertebrate limbs to maintain locomotor efficiency while coping with increasing mechanical demands of large body size. Functionally, larger animals maintain similar peak stresses to smaller animals by adopting more upright postures and limiting joint excursions during habitual motions^21,22,30–32^. More extended joint postures reduce bending stresses acting on limb bones and the necessary forces that must be generated by muscles to support limb joints^21,22,30–32^. Externally-derived bending stresses in any given joint posture will also be reduced by shortening segment length, which may be a selective pressure driving stronger negative allometric signals in more distal limb segments (Fig. 2; Supplementary Data 1–14; Supplementary Figs. 1 and 2). Biomechanical models have suggested that more distal limb segments and their connective joints may have lower safety factors in terms of peak muscle forces^21,22,30,31^ and bending stresses^21,22,32^. The fact that larger animals appear to scale more negatively than smaller animals in distal segments perhaps suggests that, as a first approximation, this proposed selective response is broadly continuous in nature across the body size range exhibited by tetrapods, particularly for quadrupedal striding taxa (Fig. 2; Supplementary Data 1–14; Supplementary Figs. 1 and 2). However, more proximal segments in the largest tetrapods seemingly scale either near-isometrically or with positive allometry at larger sizes. Relative lengthening of these more robust segments (that might logically be assumed to have inherently higher safety factors) may represent a compensatory mechanism to maintain stride length at more extended joint postures and thus minimise the cost of locomotion by minimising a reduction in overall limb length at larger body sizes (Fig. 2; Supplementary Data 1–14; Supplementary Figs. 1 and 2).

### (Hypothesis 3) Tetrapods occupying similar trophic ecologies have evolved towards similar body sizes

The influence of body size on the directionality of macroevolutionary radiations and as a constraint on morphological diversity is an area of long-standing interest in evolutionary biology^33–40^ and it has often been inferred that animals will tend to evolve towards a mechanically or physiologically optimum body size for a given ecological niche^33–40^. To test the hypothesis that taxa with similar trophic ecology have evolved under similar selection pressures for overall body size, we investigated the fit of seven different models of body size evolution, ranging from single-rate Brownian Motion (BM) to multi-regime Ornstein-Uhlenbeck (OU) models (OUMA/OUMVA; see Methods for details). The best fitting models are OU models (either OUMA or OUMVA, Supplementary Data S56), indicating some selection towards different body sizes for taxa with different trophic ecologies (Fig. 3). Carnivores generally have slightly higher values for body size long-term mean (θ), but much higher values for selection strength (ɑ) compared to other dietary groups, where ɑ is generally close to zero, indicating that any evolution towards a particular body size in these ecologies was minimal (Fig. 3). Collectively, these results therefore provide support for Hypothesis 3 in carnivores, but relatively weak support for other trophic groups.

These findings contrast somewhat with previous analyses of trophic ecology and body size evolution. The association between herbivory and large body size has been noted in mammals^34,35^, dinosaurs^36^ and lizards^37^, and is often explained by adaptive mechanisms like the Jarman-Bell Principle^38^ and the abundance-packet size hypothesis^39^. Quantitative tests of patterns in body size evolution have largely been restricted to mammals, where analyses across different taxonomic and temporal scales have generally concluded that herbivores evolve towards larger body sizes, which exceed those of carnivores and (when analysed) other dietary groups^34,35^. Our results suggest that such a pattern may not be ubiquitous to tetrapods as a whole. Strong selection for relatively large body size in carnivores may provide a long-term competitive advantage by increasing the accessible size range of prey^40^.

### (Hypothesis 4) Aquatic and fossorial ecologies are associated with relatively smaller limbs and greater gleno-acetabular distances

**Fig. 4:**
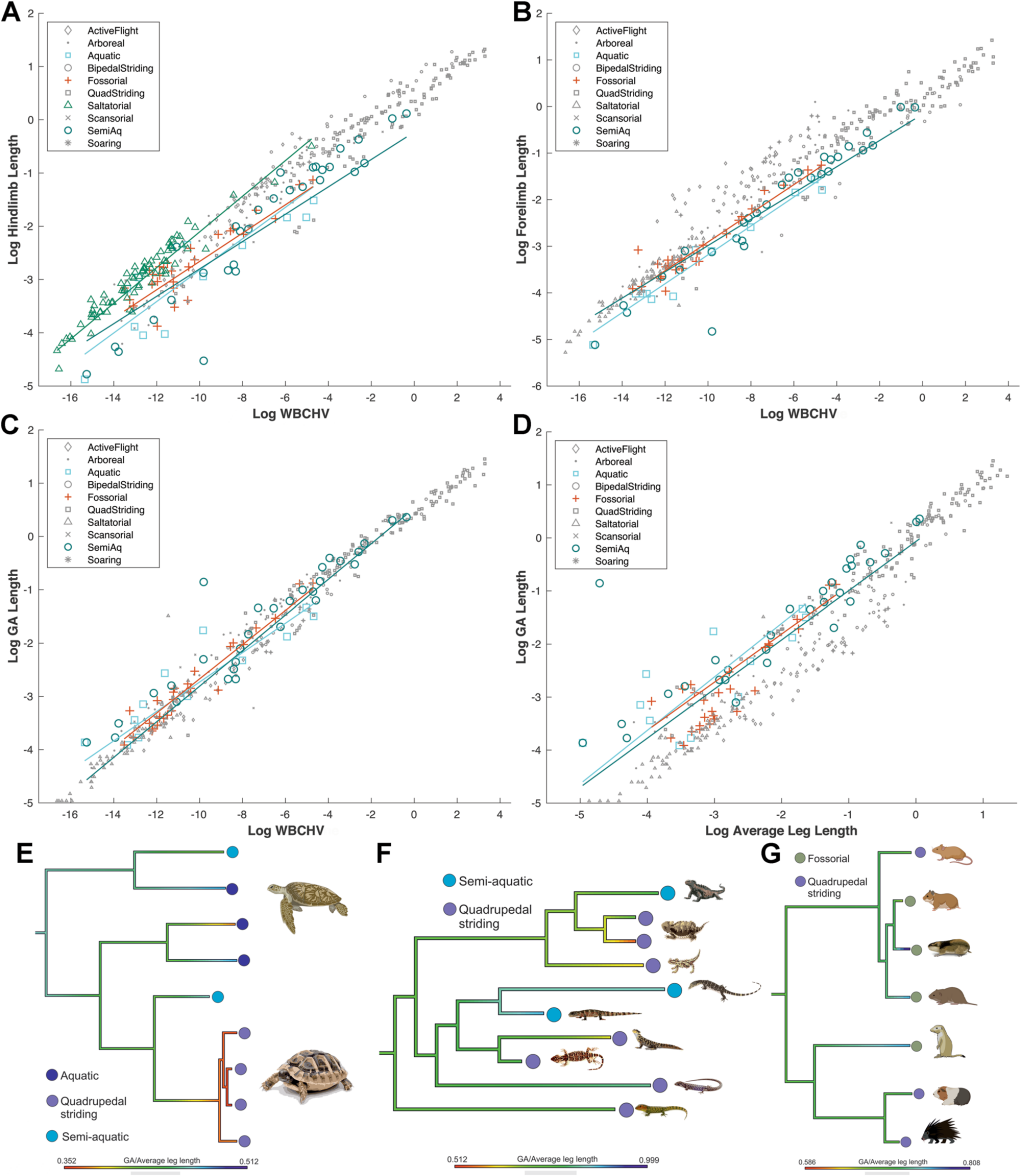
Relative limb and torso lengths in tetrapods. **A**–**G** Limb reduction and torso elongation in aquatic, semi-aquatic and fossorial tetrapods (Hypothesis 4), and **A** elongate hind limbs in saltatorial tetrapods (Hypothesis 7). Phylogenetic-informed regression provides support for relatively small **A** hind limbs and **B** forelimbs, and **C **large GA distance relative to overall size and particularly **D** average limb length in these locomotor groups. This tendency towards reduced limbs and an elongate torso can be seen within major taxonomic sub-groups that contain aquatic, semi-aquatic and fossorial species, including **E** Testudines (turtles and tortoises), **F** lizards and **G** rodents. WBCHV, whole-body convex hull volume. Source data are provided as a Source Data file. Animal images created with BioRender.com.

Changes in body shape, such as a relatively greater gleno-acetabular distances and reduced limbs, might confer obvious functional advantages for animals that habitually move through water and/or burrow through soil. Short limbs increase the capacity of limb muscles to produce backward thrust and propel the body forward and reduce drag forces^41,42^. Limb reduction, particularly of the hind limb, may be similarly beneficial for locomotion through soil. Aquatic and semi-aquatic species have the smallest hind limb and forelimb segments and overall limb lengths relative to body size and show among the greatest negative allometry and are regularly statistically different from other groups in phylANCOVAs in these respects (Fig. 4, Figs. S3–4; Supplementary Data 15–45). Fossorial taxa do have significantly shorter hind limb segments than many other non-aquatic locomotor categories (Fig. 4, Figs. S3–4; Supplementary Data 15–S20, S27), but fewer differences in the forelimb, which may be consistent with the need to maintain a relatively longer forelimb for burrowing. Flying and saltatorial groups are recovered with the longest hind limb lengths (see Supplementary Information for discussion, including evolutionary patterns, Supplementary Figs. 8–11).

Aquatic, semi-aquatic and fossorial taxa might be expected to have greater GA distances (a proxy for a more elongate body shape) for more streamlined motion through water and soil. Overall, we find partial support for this hypothesis. These locomotor modes contain individual taxa with the largest relative GA lengths (e.g*., Mustela erminea, Amphiuma means, Cryptobranchus alleganiensis*) when regressed against overall body size and limb lengths and are statistically different to certain other locomotor groups (Fig. 4, Figs. S3–4; Supplementary Data 15, S25, S30). This is particularly so for semi-aquatic taxa that have significantly longer GA than seven other locomotor categories relative to average limb length and two categories relative to overall body size (Fig. 4, Figs. S3–4; Supplementary Data 15, S25, S30). Recovery of more widespread statistical differences is likely impacted by sample size and the restriction of these categories to small body sizes.

Examination of allometric patterns recovered for GA distance across other locomotor categories also reveal several other trends that are consistent with enhanced structural support. Striding quadrupeds display significant negative allometry (Supplementary Figs. 3 and 4; Data S15), which may provide mechanical benefits by minimising increasing bending moments in the cantilevered spine as body size increases. In contrast, active fliers demonstrate the highest positive allometry in GA distance (Supplementary Figs. 3 and 4; Data S15), which may yield a change in torso shape that reduces whole-body drag through the air. Scansorial taxa also show relatively large GA lengths (Supplementary Figs. 3 and 4) and are statistically different to a number of locomotor groups (Supplementary Data 15, S25). In scansorial locomotion, a relatively large GA length may assist in maximising the proximity of torso mass to the substrate during climbing.

### (Hypothesis 5) Flying and arboreal ecologies are associated with a long forelimb

**Fig. 5:**
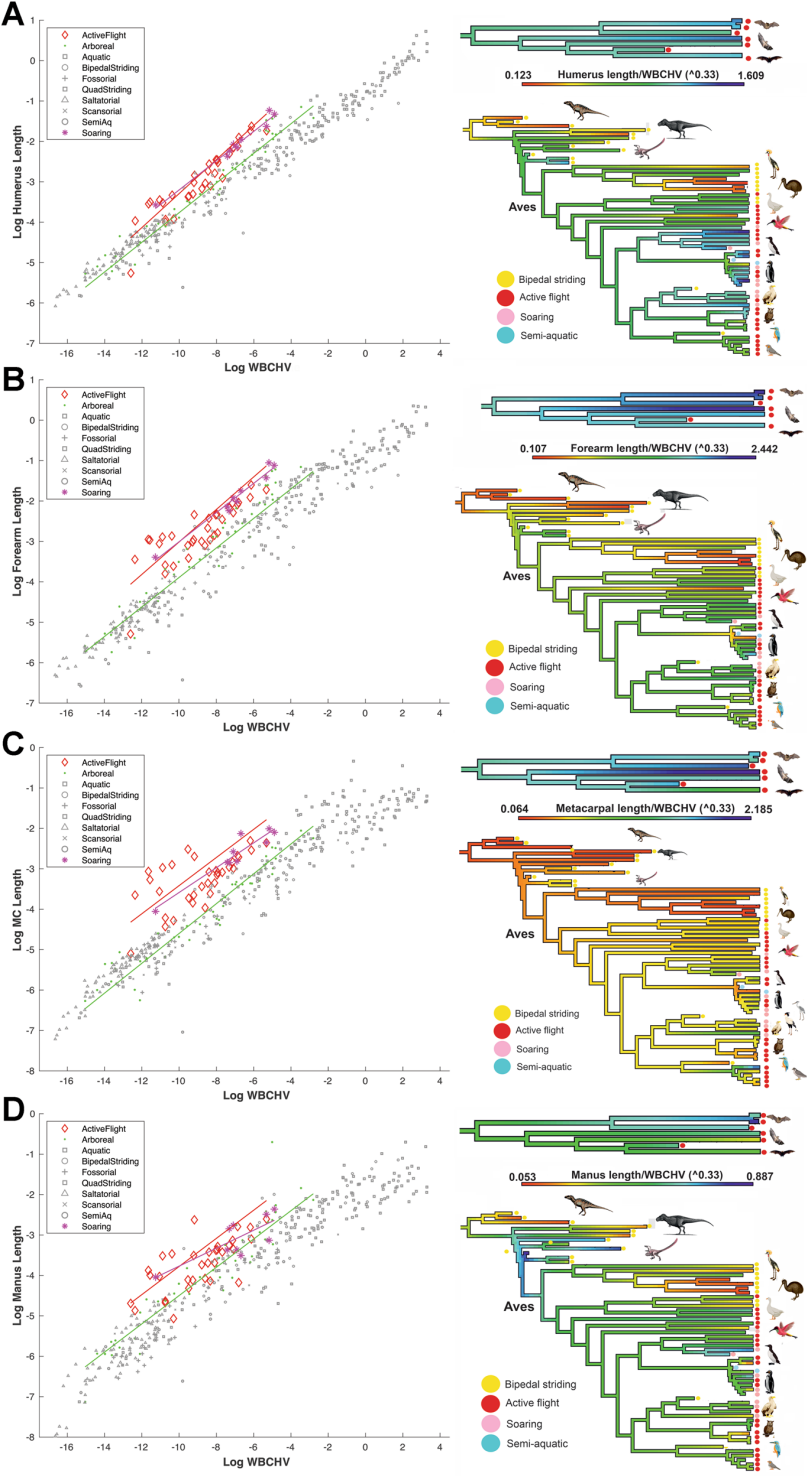
Forelimb lengths in tetrapods. Forelimb elongation in flying and arboreal taxa (Hypothesis 5). Arboreal taxa have relatively long forelimb segments and are statistically different to several other locomotor groups. Active and soaring fliers are statistically supported as having the longest **A** humeral, **B** forearm, **C** metacarpal and **D** manus segment lengths of all locomotor categories. Both groups also show similar positive allometry in proximal limbs segments (**A**, **B**) that are statistically greater than most other locomotor categories but differ from each other in the allometry of distal forelimb segments (**C**, **D**). Colour-shaded phylogenetic trees to the right of each regression graph show the evolution of forelimb segment proportions in bats and across the non-avian to avian theropod transition using ancestral state reconstruction to highlight the nature and timing of the evolutionary acquisition of enlarged forelimbs relative to body size. WBCHV, whole-body convex hull volume. Source data are provided as a Source Data file. Animal images created with BioRender.com.

Forelimb elongation may be mechanically beneficial to flying taxa by facilitating increases to wing area and muscle mass to produce both lift and thrust for take-off and sustained flight^43,44^, and to assist climbing in arboreal taxa^45,46^. Active fliers and soarers have significantly larger humeral, forearm and metacarpal segments and overall forelimb lengths than almost all other locomotor categories (Fig. 5, Figs. S3–4; Supplementary Data 15, S21–S23, S26). Manus lengths are also larger on average in flying taxa, but differences with other locomotor groups are not always statistically significant (Fig. 5, Figs. S3–4; Supplementary Data 15, S24). Flying groups also show the strongest positive allometry in proximal forelimb segments and these are significantly different to most other groups (Fig. 5, Figs. S3–4; Supplementary Data 15, S21–S23, S26). Soarers and active fliers have broadly similar overall body size ranges and show similar humeral and forearm lengths at their largest overall body sizes, but active fliers have smaller humeral lengths at smaller body sizes, which is reflected in the statistically significant differences in their PGLS slopes and intercepts (Fig. 5, Figs. S3–4; Supplementary Data 15, S21–S22). Visual inspection of reconstructed ancestral state values suggests that forelimb elongation in the dinosaurian ancestors of flying birds appears to have initiated in dromaeosaurs (*Microraptor*, *Velociraptor*), with the earliest taxa hypothesised to have had modest powered flight capabilities^47^ (*Yixianornis, Archaeopteryx*) showing overall forearm and forelimb segment sizes that are similar to extant flying birds (Fig. 5 and Supplementary Figs. S3–4, S8).

phylANCOVAs also provide support for Hypothesis 5 in arboreal taxa. Specifically, we recover significantly greater positive allometry in arboreal tetrapods compared to all non-flying locomotor groups, meaning that larger bodied arboreal tetrapods have relatively longer forelimb lengths. This contrasts with relatively strong negative allometry in the hind limb (Supplementary Fig. 3; Supplementary Data 15, 17–29), highlighting the different mechanical roles of the limbs in forelimb-driven locomotion through trees^46^.

### (Hypothesis 6) Quadrupedal striding and herbivorous ecologies are associated with relatively large torsos and forelimbs

The evolution of quadrupedality from a bipedal ancestor is rare in tetrapod evolutionary history but occurred on four independent occasions within herbivorous dinosaurs; three times within Ornithischia and once within sauropodomorphs^18,48^. Previous studies have speculated that quadrupedality evolved as the torso enlarged to increase gut size and facilitate mega-herbivory. Enlargement of the torso may have resulted not only in increased body mass but also in a craniad shift in the centre of mass, requiring longer forelimbs to provide anti-gravity support^18,48^.

**Fig. 6:**
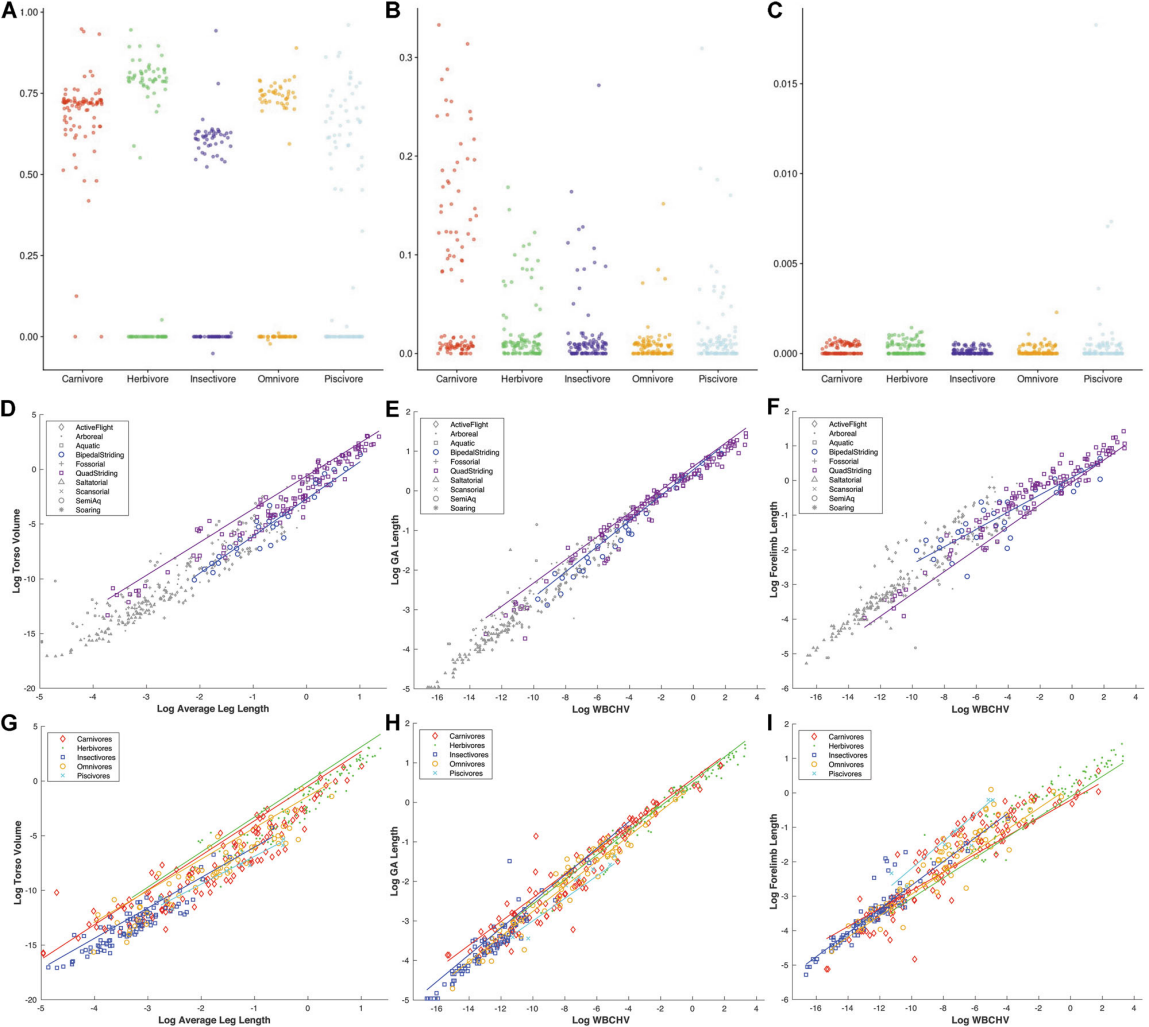
Evolution and scaling of torso and forelimb lengths in tetrapods. Relative torso and forelimb size in quadrupedal striding and herbivorous taxa (Hypothesis 6). Results for the OUwie analysis for normalised torso volume in dietary categories, showing estimates of **A** macroevolutionary optimum (θ), **B** selection strength (ɑ) and **C** evolutionary rate (Hypothesis 3). In all three panels, each point corresponds to the parameter estimate for one of the sampled simulated evolutionary regimes. For all trophic regimes across tetrapods generally, the best fitting models for the torso were OU models, indicating some selection towards different torso volumes for taxa with different trophic ecologies. Consistent with Hypothesis 6, herbivores have higher long-term mean (θ) torso volume compared to other trophic ecologies. Insectivores had the lowest θ values, whereas piscivores show high uncertainty regarding the long-term mean. Carnivores are indistinguishable from omnivores, insectivores and piscivores in terms of θ. Allometric patterns support relatively large torso sizes and GA distances in **D**, **E** quadrupeds and **G**, **H** herbivores, supporting Hypothesis 6. However, contra to Hypothesis 6, these groups have relatively short forelimbs (F&I). WBCHV, whole-body convex hull volume. Source data are provided as a Source Data file.

Our models of trait evolution and allometric analyses provide strong support for larger torsos in herbivorous taxa (Fig. 6). For all trophic regimes across tetrapods generally, the best fitting models for the torso are OU models (either OUMA or OUMVA; Data S57), indicating selection towards different torso volumes for taxa with different trophic ecologies. Consistent with Hypothesis 6, herbivores had higher long-term mean (θ) torso volumes compared to other trophic ecologies, although θ values for omnivores overlap with herbivores and with carnivores (Fig. 6). Consistent with these patterns, quadrupedal striding and herbivorous taxa had larger torso volumes for their size, and herbivores show greater positive torso allometry than all other trophic groups (Fig. 6 and Supplementary Data 46–47, S51).

Modification of forelimb use from varied types of environmental manipulation to obligatory anti-gravity support might be predicted to result in enlarged forelimbs in quadrupedal striding taxa. However, quadrupedal striding taxa have relatively short individual segments and forelimbs overall for their size compared to many other locomotor categories, including bipeds (Fig. 6). Similarly, contrary to Hypothesis 6, we found that herbivores do not show the highest long-term mean (θ) values for forelimb and segments measurements when compared to all other dietary guilds, ranking differently depending on the segment (Supplementary Figs. 5 and 6). Conversely, carnivores show the highest long-term mean (θ) value for total forelimb length, though there is no difference for total and individual segment volumes. Carnivores are also undergoing the strongest selection for both linear and volume measurements in all segments, and for total forelimb length and volume (Figs. S5–6).

**Fig. 7:**
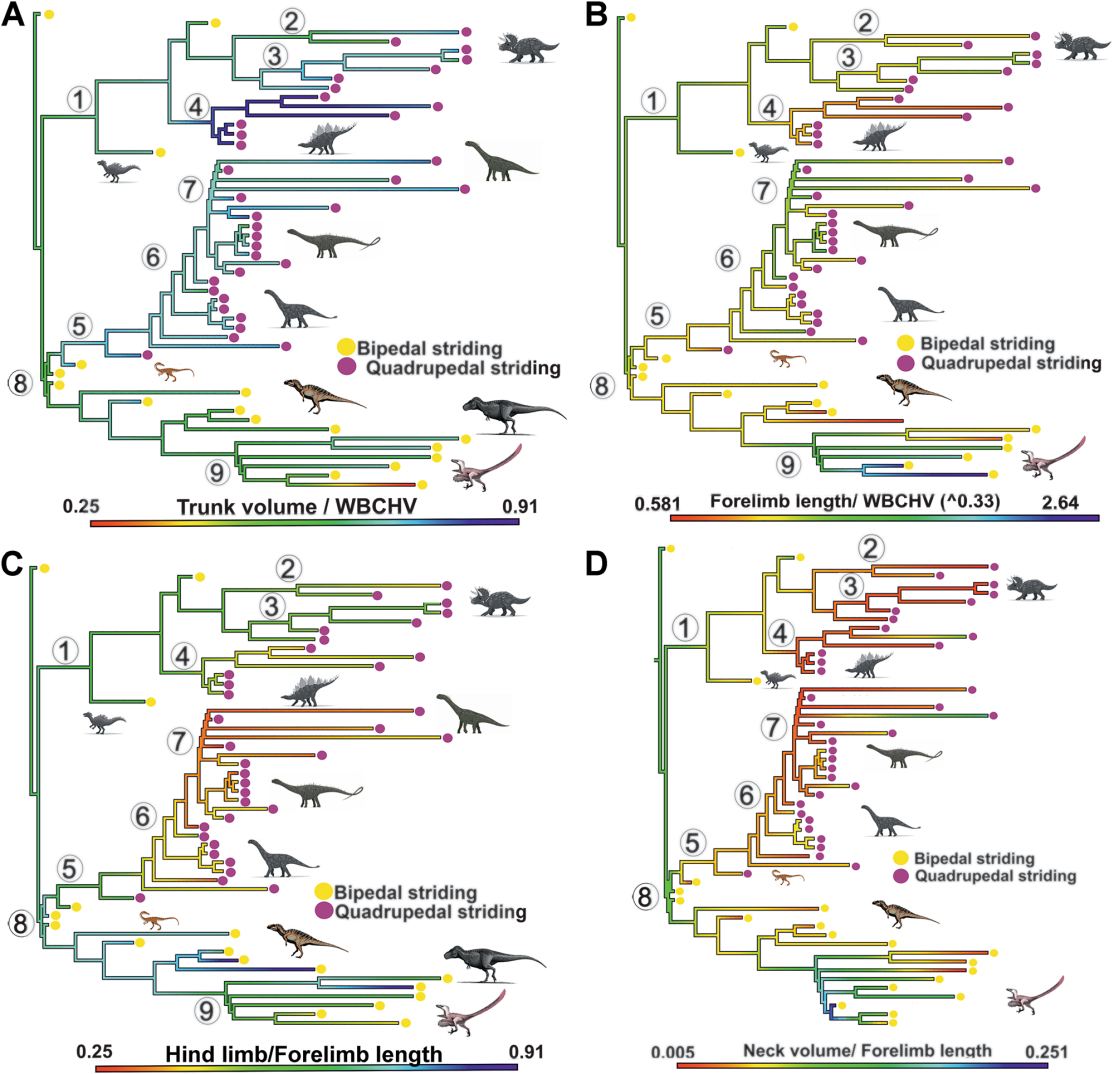
Body proportions and quadrupedality in dinosaurs. Ancestral state reconstructions of **A** trunk volume, **B** forelimb length, **C** hind limb to forelimb length ratio and **D** neck to forelimb length ratio during the transitions to quadrupedality in Dinosauria. The patterns seen during the independent acquisition of quadrupedality in ornithischians and sauropods in the relative proportions of the torso and forelimb mirror the wider allometric patterns seen in quadrupeds and herbivores generally (Fig. 6, Hypothesis 6). Relatively short forelimbs in quadrupedal dinosaurs may relate to coupling of forelimb and neck lengths to maintain the ability to graze near ground level. This is indirectly supported by the narrow and uniform range of neck to forelimb length ratio observed across quadrupedal dinosaurs with very different overall body proportions (**D**). Circled numbers represent: 1 = Ornithischia; 2 = Ceratopsia; 3=Ornithopoda; 4 = Thyreophora; 5 = Sauropods; 6 = Neosauropoda; 7= Titanisauriformes; 8 = Theropoda; 9 = Eumaniraptora. WBCHV, whole-body convex hull volume. Source data are provided as a Source Data file. Animal images created with BioRender.com.

Our data allows us to visualise how broad-scale trends in torso and limb proportions across tetrapods may also be differentially expressed during the independent evolution of quadrupedality in discrete dinosaur lineages (Fig. 7). As seen across tetrapods generally, the shift in forelimb use to active locomotory support does not appear to be universally associated with its elongation relative to overall body size across quadrupedal dinosaurs (Fig. 7B). Indeed, within striding quadrupeds only the fan-throated lizard (*Sitana ponticeriana*) has a shorter relative forelimb length than thyreophorans (Ankylosauria, Stegosauria), which have among the shortest forelimbs, relative to overall body size, of any tetrapod in our data. This raises the possibility that relative forelimb length may have decreased during the acquisition of quadrupedality in some ornithischian clades (Fig. 7B). As a result of these trends, and stronger negative allometry in hind limb length within sauropods, quadrupedal dinosaurs also exhibit noData differences in their ratios of hind limb to forelimb length, with relatively equal lengths in ornithischians, but, at least visually, a clear progressive trend towards relatively longer forelimbs than hind limbs during sauropodomorph evolution (Fig. 7C). This shift in relative limb proportions in sauropod dinosaurs may be causatively linked to neck enlargement in sauropods and a craniad shift in centre of mass position^18^, which in turn has been linked to changes in locomotion and environmental distributions in the Late Jurassic and Early Cretaeceous^49^. By greatly increasing the feeding envelope accessible by the head, neck elongation in sauropods may also have released modular constraints on body shape, particularly forelimb length, present in other quadrupeds. Similar modularity in neck and limb length has been suggested in extant birds^50^. It is possible that short forelimbs in thyreophorans (Fig. 7), and herbivores generally (Fig. 6), could be related to the need to reach the ground to graze, and this is indirectly supported by the narrow and uniform range of neck to forelimb length ratio observed across quadrupedal dinosaurs with very different overall body proportions (Fig. 7D).

We also recover previously unrecognised variability in relative torso volume across bipedal and quadrupedal dinosaurs (Fig. 7A). While all quadrupeds appear to have relatively larger torsos for their size compared to bipedal taxa, we find ceratopsians (Marginocephalia), and hadrosaurids (Ornithopoda) have much smaller torsos than sauropods and particularly thyreophorans (Fig. 7A). Greater relative torso size is seen in thyreophorans combined with short forelimbs for their body size relative to other quadrupedal dinosaurs (Fig. 7A, B) and quadrupeds generally, is likely to have negatively impacted on locomotor performance, perhaps limiting their ability to outrun predators. It is possible that extensive dermal armour evolved in concert with body proportion changes to provide active or passive predator defense, given (all other things being equal) this additional mass is likely to have reduced locomotor performance. In contrast, hadrosaurids, which lacked the more elaborate dermal and cranial ornamentation seen in marginocephalians and thyreophorans, retained more cursorial limb proportions and overall limb size and a smaller torso, and presumably therefore superior locomotor performance to outrun predators^48^.

### (Hypothesis 7) Saltatorial taxa are associated with long hind limbs as a result of relatively large distal segments

**Fig. 8:**
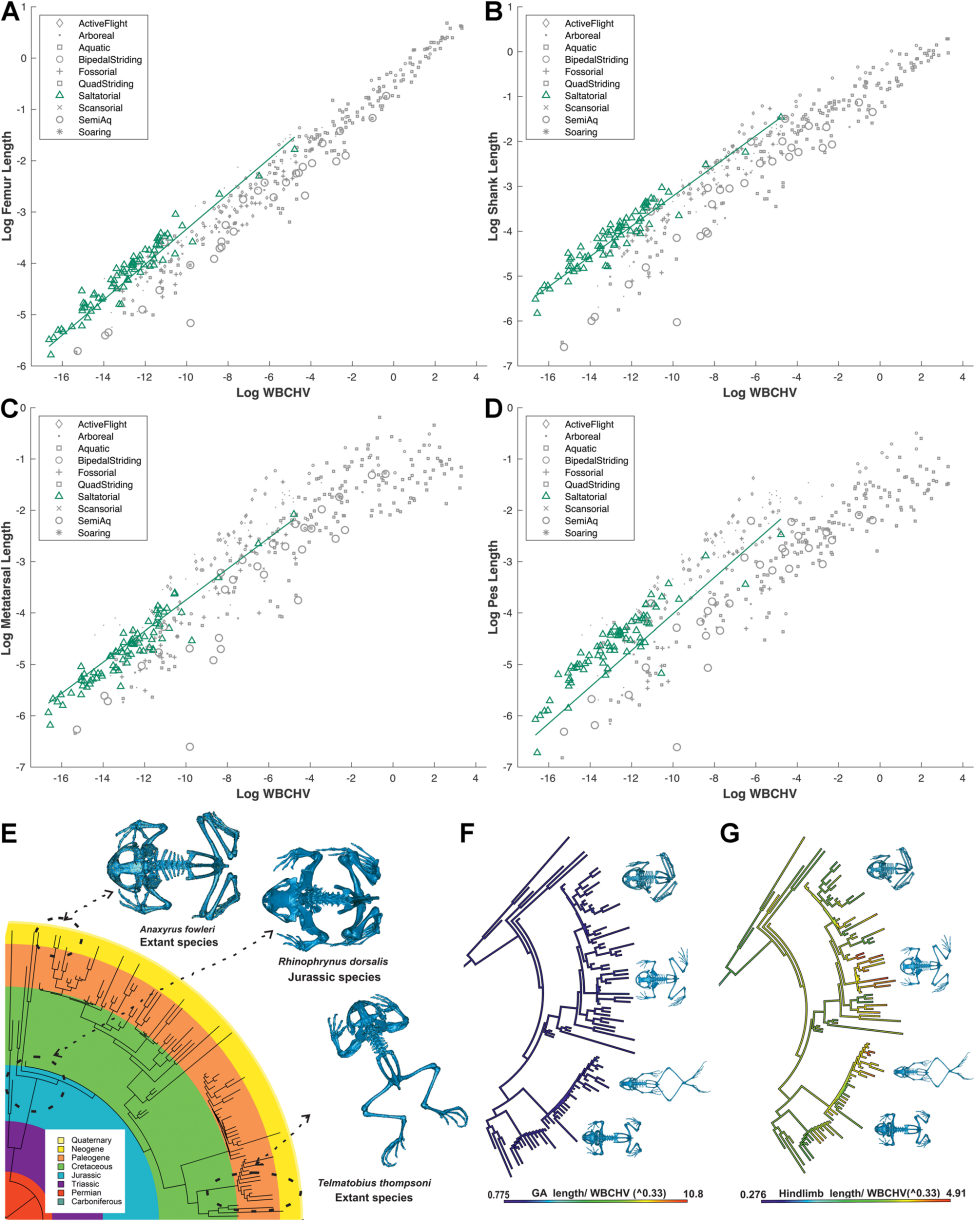
Body proportions in saltatorial tetrapods. Phylogenetic-informed regression provides support for relatively long **A** femur **B**, shank **C** metatarsal and **D** pes segment lengths in saltatorial taxa compared to most other tetrapod groups. Our saltatorial group is dominated by anurans, which have been hypothesised to have been conservative in their overall body proportions since the Triassic, which is qualitatively consistent with **E**–**G** visual trends in ancestral state values recovered here. This apparent conservatism underpins the low levels of variability (e.g. coefficients of variation; Supplementary Data 63-84) seen in limb and body proportions in saltatorial taxa. This contrasts with much greater variability in distal limb segments seen in other locomotor groups, which may reflect the need for distal limb segments to interact directly with disparate environments in a range of locomotor and non-locomotor functions that carry varied mechanical demands. WBCHV, whole-body convex hull volume. Source data are provided as a Source Data file.

Biomechanical simulations of jumping have demonstrated that longer legs enable an animal to accelerate over a greater distance, meaning that limb extensor muscles have a longer time to shorten (increasing force output) and thus to accelerate the animal to a given speed^51^. This is consistent with anecdotal observations that many specialist jumpers, like bushbabies and frogs, have elongated distal segments^51^. Consistent with Hypothesis 7, we find that saltatorial species are statistically supported as having the longest hind limbs (Fig. 4A, Data S27) and significantly longer femur and shank segments relative to body size than all other locomotor categories (Fig. 8A, B; Data S17–18). However, contra to Hypothesis 7, while the mean metatarsal and pes lengths of saltatorial taxa are higher than most locomotor categories (Fig. 8C, D), they are not statistically significantly longer (Supplementary Data 19–20), and in fact they appear to possess relatively shorter metatarsal segments than active fliers (Data S19). It appears therefore that elongate hind limbs in saltatorial taxa are primarily the result of relatively long proximal segments (femur, shank). However, the saltatorial category is dominated taxonomically by anurans, which may influence these findings. Indeed, the saltatorial category displays relatively modest variation visually in hind limb segment lengths (Fig. 8A–D), which is supported by very low values of co-efficient of variation (CoV) in all size-normalised limb and axial segment properties relative to other locomotor groups (Supplementary Data 63–84). These results are qualitatively consistent with the hypothesis put forward previously that the body proportions of saltatorial anurans have been relatively fixed since the Triassic^52,53^, with relatively elongate limbs and the reduction of trunk vertebrae^52^ leading to a relatively short GA length (Fig. 8E, F). Much greater variation in the distal limb segments of other locomotor groups potentially relates to their more varied ecological function: while the basic mechanical role of proximal limb segments remains relatively uniform across tetrapods, distal limb segments are required to interact directly with disparate environments in a range of locomotor and non-locomotor functions that carry varied mechanical demands. Across tetrapods the CoV values for distal hind limb (metatarsal and pes) and forelimb (metacarpal and manus) segment lengths and volumes were higher than more proximal segments, with magnitudes generally increasing proximally to distally (Supplementary Data 63–84). This proximal to distal gradient in CoV was regularly, but not always maintained within locomotor categories. Across tetrapods as a whole, CoVs were higher in the forelimb than the hind limb, consistent with more the varied or disparate functional role of the forelimb across locomotor and dietary ecologies (see Hypotheses 4–6). Quadrupedal striders show similar magnitudes of variation in their forelimbs and hind limbs, while bipedal taxa show considerably higher variability in the forelimb, which may be indicative of reduced constraint on segment proportions in the absence of an obligatory weight-bearing role. These trends suggest that more formal statistical tests of segment size variability may provide insight into ecomorphological patterns of limb segment evolution across tetrapods.

### (Hypothesis 8) Carnivorous and herbivorous ecologies are associated with opposing trends in head and neck size

The vertebrate head is responsible for the manipulation and consumption of food, while the neck is required to manoeuvre and stabilise the head in this and other functions. We hypothesise that, across terrestrial tetrapods, carnivores will tend to have larger heads, favouring a relatively bigger gape and stronger bite force with which to subdue large struggling prey and reduce food mass^54,55^. This relatively larger head may necessitate a shorter neck to minimise the first mass moment of the head in carnivores. In contrast, we hypothesise smaller head sizes in herbivores (as food processing shifts to the gut), and instead longer necks to increase the feeding envelope and total range of motion accessible to the head-neck system.

**Fig. 9:**
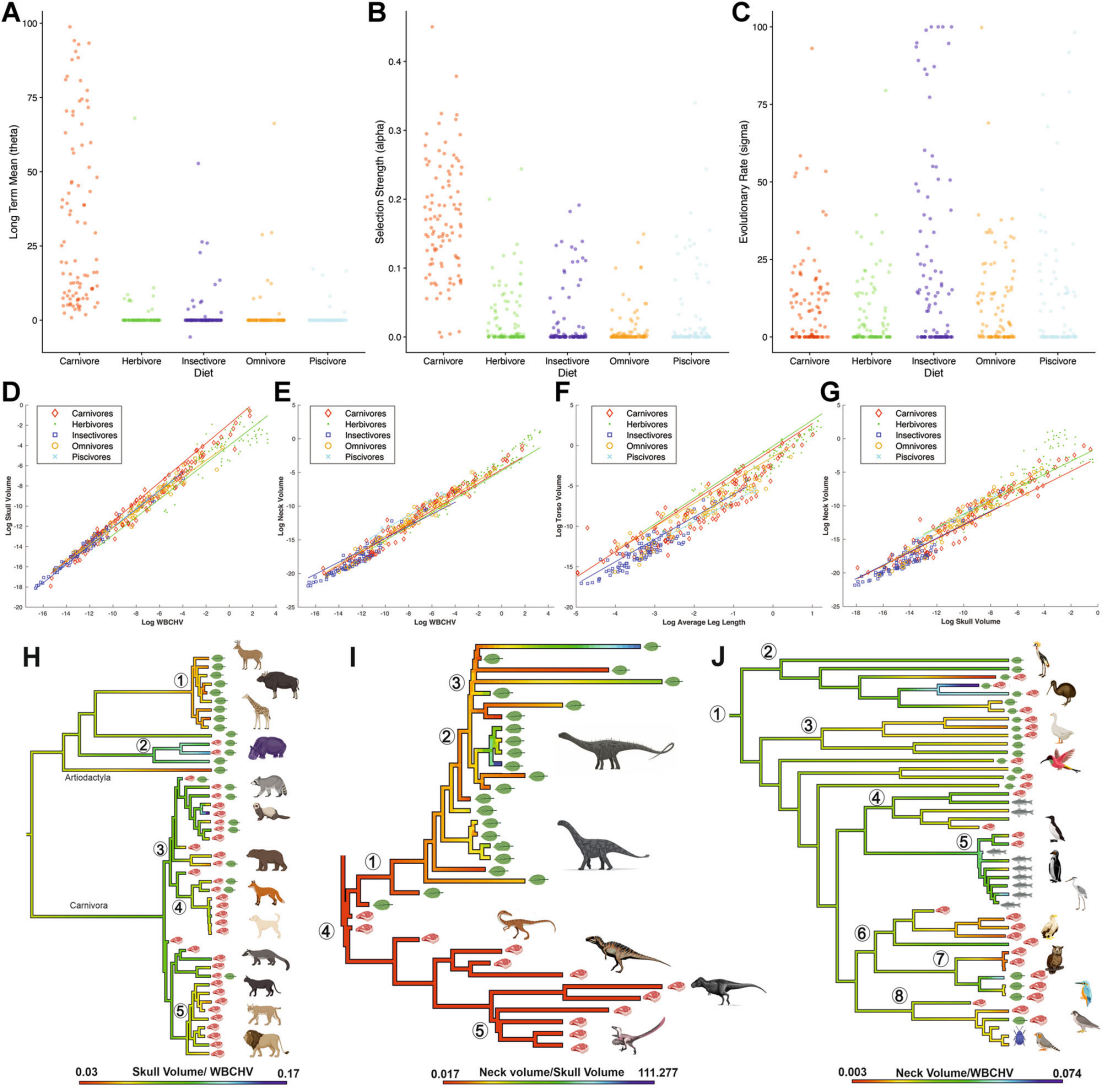
Evolution and scaling of relative head, neck and torso size in herbivorous tetrapods. Results for the OUwie analysis for head to neck ratio evolution with respect to dietary ecology, showing estimates of **A** macroevolutionary optimum (θ), **B** selection strength (ɑ) and **C** evolutionary rate (Hypothesis 3). In all three panels, each point corresponds to the parameter estimate for one of the sampled simulated evolutionary regimes. Consistent with Hypothesis 8, carnivores have greater **D** head sizes relative to body size and **A**, **G** relative to neck size compared to herbivores and other trophic groups. Contra to Hypothesis 8, **E** neck size relative to body size appears to be similar between carnivores and herbivores. The tendency towards larger heads and smaller necks in carnivores compared to herbivores (particularly at larger body sizes) is reflected in evolutionary transitions between these dietary ecologies in **H** mammals, **I** dinosaurs and **J** birds. **F** Carnivores and herbivores have larger torso volumes relative to limb lengths than other trophic groups, but do not differ from each other. Circled numbers in **H** represent: 1= Pecora; 2 = Suina; 3 = Arctoidea; 4 = Canidae; 5 = Felidae). Circled numbers in **I** represent: 1 = Sauropoda; 2 = Neosauropoda; 3 = Titanasauriformes; 4 = Theropoda; 5 = Eumaniraptora). Circled numbers in (J) represent: 1= Aves; 2 = Palaeognathae; 3= Galliformes; 4 = Aequorlitornithes; 5 = Aequornithes; 6 = Accipitriformes; 7 = Afroaves; 8 = Australaves). WBCHV, whole-body convex hull volume. Source data are provided as a Source Data file. Animal images created with BioRender.com.

The best fitting models of head, neck and head to neck ratio evolution for taxa with different trophic ecologies are OU models (either OUMA or OUMVA; Data S62), indicating some selection towards different head and neck volumes for taxa with different diets. Carnivores have higher ɑ values for head and neck volume, and head to neck ratio (Fig. 9A–C), compared to the other trophic ecologies. Although we see no evidence for differences in θ for head and neck volume across trophic ecologies, nor specifically for herbivores having smaller heads or larger necks than carnivores (Supplementary Fig. 7), carnivores do have larger relative head sizes compared to neck size than herbivores and other trophic groups (Fig. 9A–C), offering partial support for Hypothesis 8 in the macroevolutionary dynamics of these segments. However, phylANCOVAs do reveal significant differences in the allometry of these body segments between carnivores and herbivores and all other trophic groups (Fig. 9D–G; Supplementary Data 46–56) that are consistent with Hypothesis 8. Negative allometry in skull volume was recovered for all trophic groups but is least marked in carnivores, which scale closest to isometry (slope = 0.966), while herbivores scale with greatest negative allometry (slope = 0.889; Fig. 9D; Supplementary Data 47, 50). Greater selective pressure to maintain relative head size in carnivores likely relates to hyper-carnivory and large prey specialisation, particularly in the biggest carnivores. Indeed, the carnivores with the largest relative skull sizes in our dataset include frogs that specialise in eating other vertebrates of similar or larger size (*Lepidobatrachus laevis)* and reptiles, birds, dinosaurs and mammals that specialise in prey of a similar size to themselves (*Crocodilus niloticus*; *Sphenodon punctatus*; *Strix aluco*; *Falco columbarius*; *Velociraptor mongolinensis* and *Mustela erminea*).

Consistent with models of trait evolution (Supplementary Fig. 7), carnivores and herbivores are recovered with broadly similar neck volumes relative to body size, although herbivores do exhibit slightly greater positive allometry (Fig. 9E, Supplementary Data 47, 52). However (and again consistent with evolutionary models of trait evolution; Fig. 9A–C), PGLS of neck size relative to head size reveals that carnivores have significantly smaller necks for their head size than herbivores (Fig. 9D, Supplementary Data S47, S52–S53). Visual inspection of reconstructed ancestral state values for relative head and neck size across macroevolutionary transitions in diet suggests that the evolution of carnivory is marked by changes in head and neck size consistent with Hypothesis 8 (Fig. 9H–J). For example, this ecomorphological distinction is exemplified in non-avian dinosaurs: the relatively longest necks and smallest heads are found in herbivorous sauropod dinosaurs, while carnivorous theropod dinosaurs have relatively short necks and large heads for their body size relative to many terrestrial vertebrates (Fig. 9I).

### Body size, shape and ecology in tetrapods

By analysing all body segments concurrently in an extremely broad sample of extinct and extant taxa, our results provide insight into the diversity of whole-body proportions in tetrapods and its links to body size and ecology. We find statistical support for broad linear changes in body shape as size increases, but also non-linear (quadratic) patterns in quadrupedal taxa that may mechanistically balance the competing demands of safety factors and efficiency in striding gaits at the largest body sizes that have evolved in terrestrial tetrapods (Fig. 2). Models of continuous trait evolution support selection or evolution towards relatively large body sizes in carnivores (Fig. 3) and support hypothesized changes in key body segment proportions in trophic ecologies such as herbivory and carnivory (Figs. 6A–C, 9A–C; Supplementary Data 56–62). Locomotion through different media is regularly associated with differences in multiple body segments with mechanical or functional benefits; for example, smaller limbs and relatively long torsos in aquatic forms, large forelimbs in flying and arboreal taxa, and evidence for both coupled and decoupled changes in body segment proportions in quadrupedal taxa (Figs. 4–7). These findings emphasize the importance of viewing the tetrapod skeleton as a multi-modal system and analysing multiple segments concurrently to understand the nature and extent of allometric and ecological variability in vertebrate body proportions.

## Methods

### Data collection

Ethical approval was granted on 16/1/18 for the use of anonymised canine imaging data from the clinical imaging archive system of the University of Liverpool Small Animal Teaching Hospital (VREC628). Whole body scans of other specimens were obtained from a variety of existing sources including CT scans and photogrammetric models collected by the authors, as well as models from previous studies^5,12,17,18,56–61^ and online digital repositories (Morphosource, KUPRI, Digimorph, Sketchfab, animalsimulation.org). A full list of the models, including their source information, can be found in the electronic supplementary material (Supplementary Data 85). The precise ontogenetic age and sex of the majority of the specimens are unknown. However, based on overall size we infer that most of our extant specimens are adults. Body shape and size metrics were quantified from 3D skeletal models of 116 amphibians, 47 birds, 55 non-avian dinosaurs, 143 mammals, 46 non-avian reptiles and 3 reptiliomorphs spread across the major groups within these clades. The data set consisted of 318 extant and 92 extinct species.

CT data were segmented using Mimics research 20.0 (www.materialise.com/mimics) to generate 3D models of the skeletal material. Photogrammetric reconstructions were carried out in Agisoft Metashape 1.7.6 (www.agisoft.com/). The resulting surface models were processed in Meshlab 2018 (www.meshlab.net/). Any non-skeletal material was removed, and the skeleton of each specimen was split into segments (i.e., skull, neck, trunk, tail, humerus, forearm, metacarpal, manus, femur, shank, metatarsal and pes) to aid measurements and volumetric reconstructions (see below).

### Body shape and size metrics

To examine patterns of body shape variation across tetrapods in our data set we derived a variety of linear and volumetric measures of body segment proportions. The linear morphometric data collected consisted of gleno-acetabular (GA) distance, humerus length, forearm segment length, metacarpal segment length, manus segment length, femur length, shank segment length, metatarsal segment length, and pes segment length (Fig. 1). The length of the forelimbs and hind limbs were calculated by summing the lengths of the four individual segments. Measurements were taken from approximate joint centres rather than the absolute length of the bones because some species have elongated areas of bone that do not contribute to the overall segment length. The overall size of some body segments (e.g., the torso) are not well captured by a single linear measurement and we therefore also generated volumetric size metrics using convex hulls^17,18^. This yields an approximation of the skeletal volume of each individual body segment in our models, which can be summed to generate a whole-body skeletal volume (herein referred to as whole-body convex hull volume [WBCHV]).

In addition to analysing how these raw measurements varied across our data set, we also used them as a basis to calculate body shape and size-normalised metrics. For various size-normalised comparisons (e.g. Hypothesis 7) we normalised body segment linear measurements by WBCHV^0.33^ (e.g., femur length/ WBCHV^0.33^) and volumetric segment measurements by WBCHV (e.g., torso volume/ WBCHV). For regression analyses (see below; Supplementary Data 1–55) we used WBCHV as our proxy for whole body size. We preferred WBCHV as a proxy for overall body size because it uses the entire skeleton rather than relying on a measure from a single body segment, which may bias any further analyses due to potential allometric signals in that one body segment. Using WBCHV also allowed all linear and volumetric parameters to be assessed or normalised by the same size metric (e.g., if femur length were used as the body size metric then a second body size metric would be needed to be found to size-normalise femur length). Also, variability in scan/model resolution meant that popular alternative metrics (e.g., long bone circumference^23^) could not be accurately and/or repeatably measured across our data set.

### Phylogenetic statistical analysis

To analyse body proportions with phylogenetic context, each tetrapod species was added to a phylogenetic tree, which was built by merging recent, taxon-rich cladograms of major tetrapod groups using Mesquite (www.mesquiteproject.org). A full list of the phylogenetic trees merged, including their source information, can be found in the electronic supplementary material (Supplementary Data 86). To calculate branch lengths and time-calibrate the tree, first and last occurrences of each species were taken as the stratigraphic range of the formation in which the fossil was found in the Palaeobiology Database (www.paleobiodb.org). First and last occurrences used are tabulated in Supplementary Data 87. Branch lengths were calculated using the ‘equal’ method using the *DatePhylo* function within the R package *Strap*^60^ in order to avoid zero branch length values. To examine variations and correlations in body shape and ecological variables we classified taxa into locomotor and dietary categories based on information in the literature about their primary mode of locomotion and diet (Supplementary Data 88–89). The locomotor mode of each taxon was classified as either active flight (*n* = 33), aquatic (*n* = 12), arboreal (*n* = 42), bipedal striding (*n* = 32), fossorial (*n* = 28), quadrupedal striding (*n* = 131), saltatorial (*n* = 79), scansorial (*n* = 14), semi-aquatic (*n* = 30) or soaring flight (*n* = 9). The dietary ecology of each taxon was classified as either carnivore (*n* = 123), herbivore (*n* = 120), insectivore (*n* = 95), omnivore (*n* = 64), or piscivore (*n* = 9). Here we focus on locomotion and diet because of the mechanistic links between body proportions and animal mechanics and physiology^1–7^ (see introduction), and because it is possible to categorise both extant and extinct into ecological sub-groups with reasonable objectivity.

To examine how each body segment metric scaled with body size across the data set (Hypothesis 1, Supplementary Data 1 and 2) we conducted regression analyses using phylogenetic generalised least squares (PGLS) in the R package *caper*^24^. This approach follows a general linear model calculating the slope, intercept, confidence, and prediction intervals, adjusting the expected covariance according to phylogenetic relationships. We also tested if quadratic models provided a statistically better fit to scaling trends than linear fits (Hypothesis 2, Supplementary Data 3–8) in log-transformed parameter versus WBCHV data sets^23^. A statistically significant second-degree coefficient established, if present, the nonlinear nature of the data. Models were compared using Akaike weights and associated Akaike information criteria for limited sample sizes (AICc). To examine the nature of non-linearity in body segment allometry we compared the linear (PGLS) slopes of taxa within a series of size thresholds (or size bins), where the data set was split at above versus below ~25 kg body mass, above versus below ~100 kg body mass, and above versus below ~500 kg body mass (Supplementary Data 9–14). These thresholds were chosen in part based on various size-thresholds recovered in previous studies^20,27,30^ and in part because they allowed for a reasonable sample size in our largest size category. All data were log-transformed prior to these regression analyses. Pagel’s lambda (λ) was used to estimate the strength of the phylogenetic signal in the analyses. All analysis was carried out in R using the packages *qpcr*, *ape*, *GEIGER* and *nlme*^24,62–65^. Phylogenetic ANCOVAs (phylANCOVA) were used to test for differences in the allometric relationships between locomotor and dietary groups (Supplementary Data 15–55) using the approach of Smaers and Rohlf (2016)^66^. Examples of the code used for these regression analyses are provided in Supplementary Code 1–3.

To test the hypotheses (Hypotheses 3, 6 and 8) that taxa with similar trophic ecology would have evolved under similar selection pressures or evolutionary regimes, we used models of continuous trait evolution that allow evolution to vary with evolutionary regime^67^. Before fitting the models, we defined the evolutionary regime that each node of the tree belonged to. This was achieved using a Stochastic Character Mapping (SIMMAP^68^) procedure, in which we jointly estimated the most likely state at the root of the tree and average transition rates between the different states, using the function *make.simmap* from the phytools package^69^. The transition mode was chosen by selecting the best model between Equal Rates (ER), Symmetrical (SYM) and All Rates Different (ARD) by comparing the AICc value of each model (Supplementary Data 56–62). The preferred model for trophic ecology was the ER (AICc__ER_ = 985.09, AICc__SYM_ = 1330.88. AICc__ARD_ = 1213.95) and using the transition rates and root states estimated with this model we simulated 1,000 different possible evolutionary histories for trophic ecology on the tree (five states: carnivore, herbivore, insectivore, omnivore, piscivore), from which we randomly selected 100 maps/histories for the next steps. Note that ideally, we would also have simulated evolutionary regimes for locomotor modes, but the number of categories (10) and low numbers of taxa in some categories, meant the models below could not be fitted reliably, and the results were not biologically meaningful.

Next, for a given body dimension variable (e.g. body size) and trophic ecology evolutionary regime we fitted seven different models of continuous trait evolution using the package OUwie^67^. These were: 1) Brownian motion (BM) model, a random walk model with one rate of evolution (σ^2^) for all regimes; 2) single stationary peak Ornstein Uhlenbeck (OU) model, a random walk model with one rate of evolution for all regimes (σ^2^) but where evolution is towards a long-term mean (θ) with an attraction strength (ɑ); 3) multi-rate Brownian motion (BMS) model, a random walk model with different σ^2^ for each regime; 4) multi-optima OU (OUM) model, a random walk model with one σ^2^ for all regimes, with a single ɑ but where evolution is towards a different θ for each regime; 5) multi-rate multi-optima OU (OUMV) model, a random walk model with different σ^2^ for each regime, a single ɑ, and evolution towards a different θ for each regime; 6) multi-alpha multi-optima OU (OUMA) model, a random walk model with a single σ^2^, a different ɑ for each regime, and evolution towards a different θ for each regime; and 7) multi-rate multi-alpha multi-optima OU (OUMVA) model, a random walk model with different σ^2^, ɑ and θ for each regime. We fitted each model for each of the 100 randomly selected evolutionary scenarios of each discrete trait evolution. We used AIC (AICc) to identify the best fitting model(s). We present variation across the results from 100 simulated evolutionary histories (i.e. stochastic maps) of trophic ecology by plotting the raw parameter values for the best selected model for each history. However, it is not possible to directly compare parameter estimates between two different stochastic maps, due to each replica having different likelihood values. Therefore, to assess the differences in the values for the whole sample of maps, we first calculated the pairwise differences for each parameter between different trophic ecology state within each of the maps, and summarised the number of maps for which the difference patterns (e.g. rate parameter being higher for carnivores than for any other diet) were seen. This way, since we cannot access the true evolutionary history of trophic ecology, we believe we can incorporate some uncertainty on this evolutionary history that arises from limitations of the approach (e.g. estimating internal node and branches’ states using data only from the tips) as well as highlight the observed general differences between trophic ecologies. We tested the following specific combinations of body dimensions and trophic ecology evolutionary regime. (1) body size (Hypothesis 3); (2) torso volume, forelimb length and forelimb volume (Hypothesis 6); (3) hindlimb length, femur length, shank length, metatarsal length, pes length, and hindlimb volume (Hypothesis 7); and (4) head volume, neck volume and head volume to neck volume ratio (Hypothesis 8). The code used to carry out these analyses is provided in Supplementary Code 4. To visualise changes in body shape across major evolution transitions (Figs. 4–9) we used the Brownian motion model to apply ancestral state estimation using the ‘*phytools’* function ‘*contmap*’.

## Supplementary information

Supplementary Information

Description of Additional Supplementary Files

Supplementary Data 1-89

Supplementary Code 1-4

## Data Availability

3D volumetric models, code and numerical data generated in this study have been deposited in the University of Liverpool’s Research Data Catalogue (10.17638/datacat.liverpool.ac.uk/1733). Statistical outputs from the data are provided in the Supplementary Data. All data necessary for recreating the figures are available in the Source Data file.

## Code availability

All scripts required to repeat the statistical analyses are available in the Supplementary Code files and from the University of Liverpool’s Research Data Catalogue (10.17638/datacat.liverpool.ac.uk/1733).

## References

[1] Cuvier, G. L. C. F. D. Le règne animale. *Deterville, Paris*. (1817).

[2] Russell, E. S. *Form and function: A contribution to the history of animal morphology*. J. Murray. (1916).

[3] Lauder GV (1981). Form and function: structural analysis in evolutionary morphology. Paleobiology.

[4] Rushton JP, Rushton EW (2004). Progressive changes in brain size and musculo-skeletal traits in seven hominoid populations. Hum. Evolution.

[5] Clauss M, Steuer P, Müller DW, Codron D, Hummel J (2013). Herbivory and body size: allometries of diet quality and gastrointestinal physiology, and implications for herbivore ecology and dinosaur gigantism. PLoS One.

[6] Phillips PK, Heath JE (1995). Dependency of surface temperature regulation on body size in terrestrial mammals. J. Therm. Biol..

[7] Henderson DM (2013). Sauropod necks: are they really for heat loss?. PloS ONE.

[8] Ebenman B (1992). Evolution in organisms that change their niches during the life cycle. Am. Naturalist.

[9] Harmon LJ, Kolbe JJ, Cheverud JM, Losos JB (2005). Convergence and the multidimensional niche. Evolution.

[10] LaBarbera, M. The evolution and ecology of body size. In *Patterns and Processes in the History of Life* 69–98 (Springer, Berlin, Heidelberg, 1986).

[11] Edgington HA, Taylor DR (2019). Ecological contributions to body shape evolution in salamanders of the genus Eurycea (Plethodontidae). PLoS ONE.

[12] Allen V, Bates KT, Li Z, Hutchinson JR (2013). Linking the evolution of body shape and locomotor biomechanics in bird-line archosaurs. Nature.

[13] Claverie T, Wainwright PC (2014). A morphospace for reef fishes: elongation is the dominant axis of body shape evolution. PloS ONE.

[14] Law CJ (2020). Evolutionary and morphological patterns underlying carnivoran body shape diversity. Evolution.

[15] Wainwright PC (1991). Ecomorphology: experimental functional anatomy for ecological problems. Am. Zool..

[16] Bhullar BAS (2012). Birds have paedomorphic dinosaur skulls. Nature.

[17] Sellers WI (2012). Minimum convex hull mass estimations of complete mounted skeletons. Biol. Lett..

[18] Bates KT (2016). Temporal and phylogenetic evolution of the sauropod dinosaur body plan. R. Soc. Open Sci..

[19] Alexander RM, Jayes AS, Maloiy G, Wathuta EM (1979). Allometry in the limb bones of mammals from shrews (*Sorex*) to elephant (*Loxodontia*). J. Zool..

[20] Economos AC (1983). Elastic and/or geometric similarity in mammalian design. J. Theor. Biol..

[21] Biewener AA (1989). Scaling body support in mammals: limb posture and muscle mechanics. Science.

[22] Biewener A (2005). A Biomechanical consequence of scaling. J. Exp. Biol..

[23] Campione NE (2017). Extrapolating body masses in large terrestrial vertebrates. Paleobiology.

[24] Orme D (2013). The caper package: comparative analysis of phylogenetics and evolution in R. R. package version.

[25] Coombs WP (1978). Theoretical aspects of cursorial adaptations in dinosaurs. Q. Rev. Biol..

[26] Christiansen P (2002). Locomotion in terrestrial mammals: the influence of body mass, limb length and bone proportions on speed. Zool. J. Linn. Soc..

[27] Silva M (1998). Allometric scaling of body length: elastic or geometric similarity in mammalian design. J. Mammal..

[28] Fuentes MA (2016). Theoretical considerations on maximum running speeds for large and small animals. J. Theor. Biol..

[29] Hirt MR, Jetz W, Rall BC, Brose U (2017). A general scaling law reveals why the largest animals are not the fastest. Nat. Ecol. Evolution.

[30] Ren L, Miller CE, Lair R, Hutchinson JR (2010). Integration of biomechanical compliance, leverage, and power in elephant limbs. PNAS.

[31] Hutchinson JR (2004). Biomechanical modeling and sensitivity analysis of bipedal running ability. I. Extant taxa. J. Morphol..

[32] Sellers WI, Pond SB, Brassey CA, Manning PL, Bates KT (2017). Investigating the running abilities of *Tyrannosaurus rex* using stress-constrained multibody dynamic analysis. PeerJ.

[33] Stanley SM (1973). An explanation for Cope’s rule. Evolution.

[34] Price SA, Hopkins SSB (2015). The macroevolutionary relationship between diet and body size in mammals. Biol. J. Linn. Soc..

[35] Smith FA (2010). The evolution of maximum body size of terrestrial mammals. Science.

[36] Benson RBJ (2014). Rates of dinosaur body mass evolution indicate 170 million years of sustained ecological innovation on the avian stem lineage. PLoS Biol..

[37] Cooper WE, Vitt LJ (2002). Distribution, extent, and evolution of plant consumption by lizards. J. Zool..

[38] Geist V (1974). On the relationship of social evolution and ecology in ungulates. Am. Zool..

[39] Olsen A (2015). Exceptional avian herbivores: multiple transitions toward herbivory in the bird order Anseriformes and its correlation with body mass. Ecol. Evolution.

[40] Wilson DS (1975). The adequacy of body size as a niche difference. Am. Naturalist.

[41] Amson E, de Muizon C, Laurin M, Argot C, de Buffrénil V (2014). Gradual adaptation of bone structure to aquatic lifestyle in extinct sloths from Peru. Proc. R. Soc. B: Biol. Sci..

[42] Fish FE (2004). Structure and mechanics of nonpiscine control surfaces. IEEE J. Ocean. Eng..

[43] Rader JA, Hedrick TL, He Y, Waldrop LD (2020). Functional morphology of gliding flight II. Morphology follows predictions of gliding performance. Integr. Comp. Biol..

[44] Tobalske BW (2007). Biomechanics of bird flight. J. Exp. Biol..

[45] Samuels JX, Van Valkenburgh B (2008). Skeletal indicators of locomotor adaptations in living and extinct rodents. J. Morphol..

[46] Selby MS, Lovejoy CO, Byron CD (2020). Odd-nosed monkey scapular morphology converges on that of arm-swinging apes. J. Hum. Evolution.

[47] Pei R (2020). Potential for powered flight neared by most close avialan relatives, but few crossed its thresholds. Curr. Biol..

[48] Maidment SCR, Linton DH, Upchurch P, Barrett PM (2012). Limb-bone scaling indicates diverse stance and gait in quadrupedal ornithischian dinosaurs. PLoS ONE.

[49] Mannion PD, Upchurch P (2010). A quantitative analysis of environmental associations in sauropod dinosaurs. Paleobiology.

[50] Bohmer C, Plateau O, Cornette R, Abourachid A (2019). Correlated evolution of neck length and leg length in birds. R. Soc. Open Sci..

[51] Alexander RM (1995). Leg design and jumping technique for humans, other vertebrates and insects. Philos. Trans. R. Soc. B.

[52] Shubin NH, Jenkins FA (1995). An early Jurassic jumping frog. Nature.

[53] Reilly SM, Jorgensen ME (2011). The evolution of jumping in frogs: morphological evidence for the basal anuran locomotor condition and the radiation of locomotor systems in crown group anurans. J. Morphol..

[54] Anderson RA, McBrayer LD, Herrel A (2008). Bite force in vertebrates: opportunities and caveats for use of a nonpareil whole-animal performance measure. Biol. J. Linn. Soc..

[55] Erickson GM, Lappin KA, Vliet KA (2003). The ontogeny of bite-force performance in American alligator (*Alligator mississippiensis*). J. Zool. Lond..

[56] Bates KT (2009). How big was “Big Al”?. Palaeontologia Electron..

[57] Bates KT, Manning PL, Hodgetts D, Sellers WI (2009). Estimating mass properties of dinosaurs using laser imaging and 3D computer modelling. PLoS ONE.

[58] Macaulay S, Hutchinson JR, Bates KT (2017). A quantitative evaluation of physical and digital approaches to centre of mass estimation. J. Anat..

[59] Schachner ER (2017). Pulmonary anatomy and a case of unilateral aplasia in a common snapping turtle (Chelydra serpentina): developmental perspectives on cryptodiran lungs. J. Anat..

[60] Schachner ER, Cieri RL, Butler JP, Farmer CG (2014). Unidirectional pulmonary airflow patterns in the savannah monitor lizard. Nature.

[61] Bell MA, Lloyd GT (2015). strap: An R package for plotting phylogenies against stratigraphy and assessing their stratigraphic congruence. Palaeontology.

[62] Andrej-Nikolai, S. qpcR: modelling and analysis of real-time PCR data, Version 1.4-0. (2014).

[63] Pennell MW (2014). geiger v2. 0: an expanded suite of methods for fitting macroevolutionary models to phylogenetic trees. Bioinformatics.

[64] Pinheiro, J., Bates, D., DebRoy, S., & Sarkar, D. R Core Team. 2015. nlme: linear and nonlinear mixed effects models. R package version 3.1-120. R package version, 3-1. (2015).

[65] Paradis E, Claude J, Strimmer K (2004). APE: analyses of phylogenetics and evolution in R language. Bioinformatics.

[66] Smaers JB, Rohlf FJ (2016). Testing species’ deviation from allometric predictions using the phylogenetic regression. Evolution.

[67] Beaulieu JM, Jhwueng DC, Boettiger C, O’Meara BC (2012). Modeling stabilizing selection: expanding the Ornstein-Uhlenbeck model of adaptive evolution. Evolution.

[68] Huelsenbeck JP, Neilsen R, Bollback JP (2003). Stochastic mapping of morphological characters. Syst. Biol..

[69] Revell LJ (2012). phytools: an R package for phylogenetic comparative biology (and other things). Methods Ecol. Evolution.

