# Supplementary material for: Body size, shape and ecology in tetrapods
Source: Nat Commun. 2022 Jul 27;13:4340. doi: 10.1038/s41467-022-32028-2 (PMC9329317; doi:10.1038/s41467-022-32028-2)
Supplement: Supplementary file 1 — Supplementary Information [file 41467_2022_32028_MOESM1_ESM.pdf]

Supplementary Information for

Body size and shape in terrestrial tetrapods

Alice E. Maher, Gustavo Burin, Philip G. Cox, Thomas W. Maddox, Susannah C.R. Maidment, Natalie H. Cooper, Emma R. Schachner, Karl T. Bates

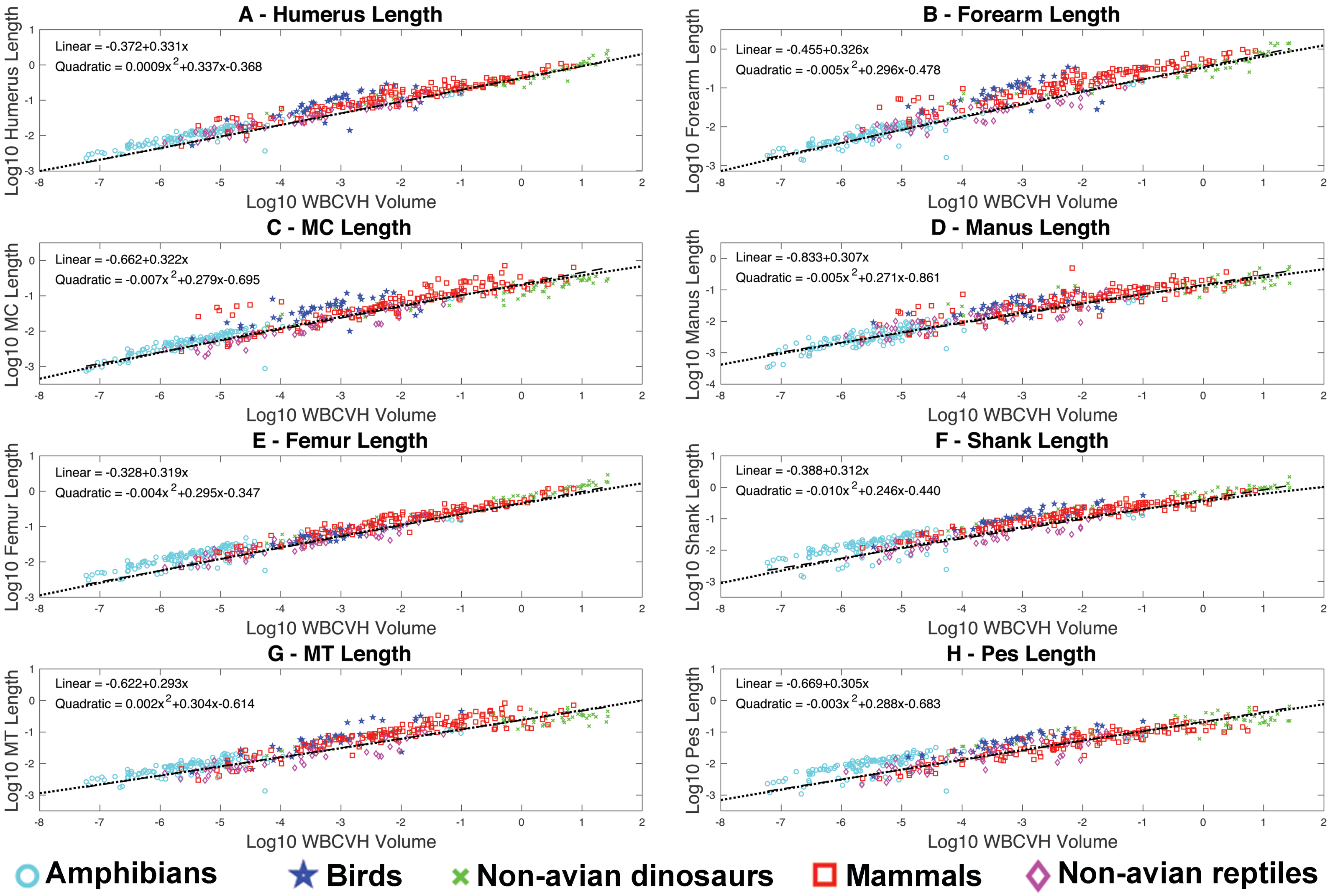

Supplementary Figure 1: Scaling of major body segments in tetrapods. Scaling relationships between major body segment size and overall body size (total whole-body skeletal convex hull volume) in 410 terrestrial tetrapods using phylogenetically informed linear (thick dashed lines) and

quadratic (thin dotted lines) fits. The (A) humerus, (B) forearm, (C) metacarpal (MC), (D) manus, (E) femur, (F) shank, (G) metatarsal and (H) pes segments are represented by lengths. Isometry in (A-C) would be a slope of 0.33. Full breakdowns of the regression model information can be found in Supplementary Data 1-14, including additional comparisons of scaling in limb segment volumes. Taxa have been colour-coded by taxonomic order for display purposes. Source data are provided as a Source Data file.

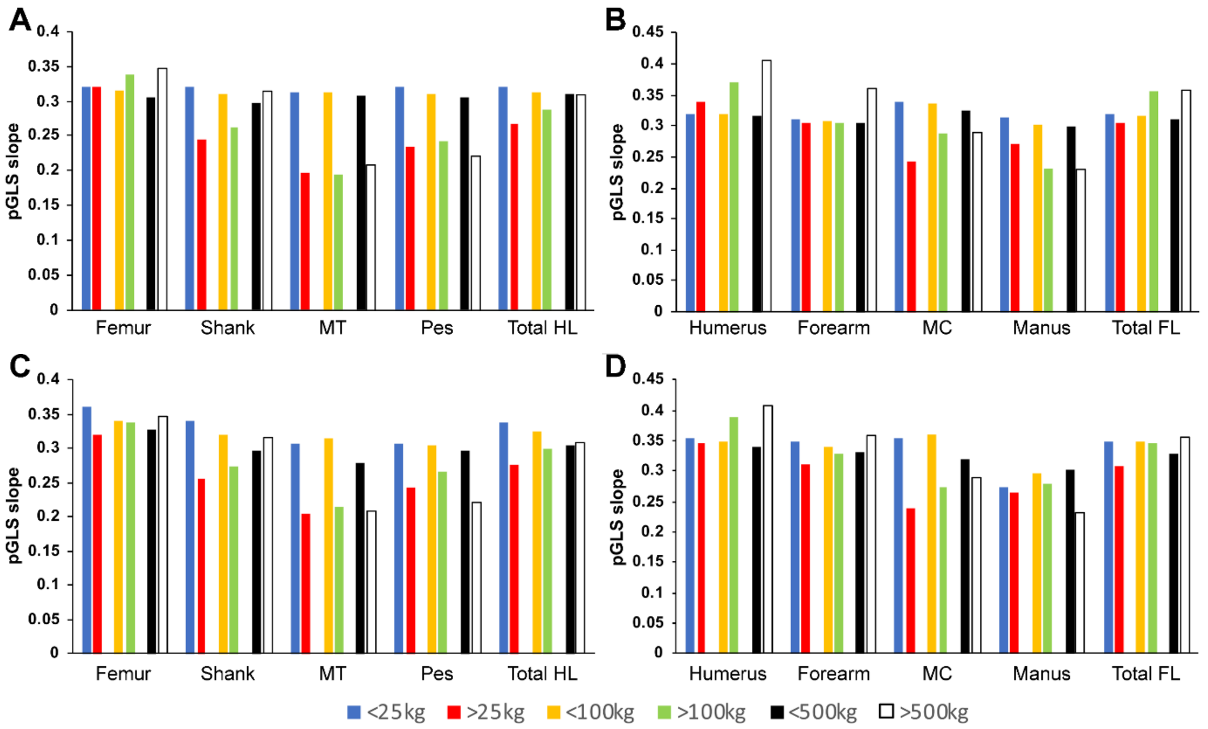

Supplementary Figure 2. Assessing differential scaling in small-to-large tetrapods. Phylogenetic generalised least squares (pGLS) regression slopes for individual body segments with taxa split into body size bins (<25kg vs. >25kg; <100kg vs. >100kg; <500kg vs. >500kg) in (A-B) the full all tetrapod data set and (C-D) quadrupedal striding taxa only. Source data are provided as a Source Data file.

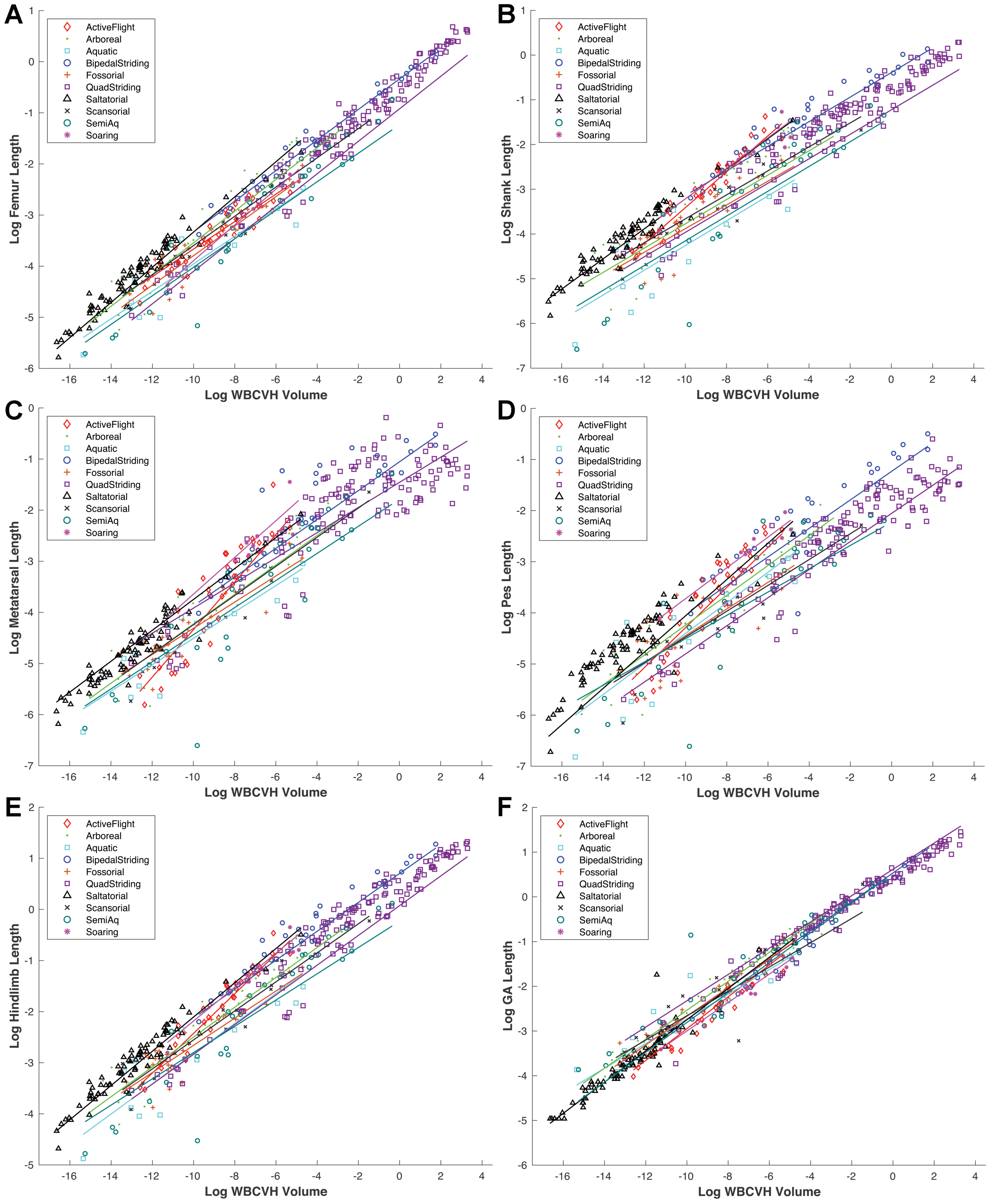

Supplementary Figure 3. Scaling in the hindlimbs different locomotor groups. Scaling relationships between hindlimb body segment length and GA length versus overall body size (total whole-body skeletal convex hull volume [WBCHV]) in different locomotor categories, with statistical differences between phylogenetically informed linear fits tested using a phlyANCOVA.

Relationships shown are (A) femur, (B) shank, (C) metatarsal, (D) pes, E) total hind limb length and (F) GA length. Source data are provided as a Source Data file.

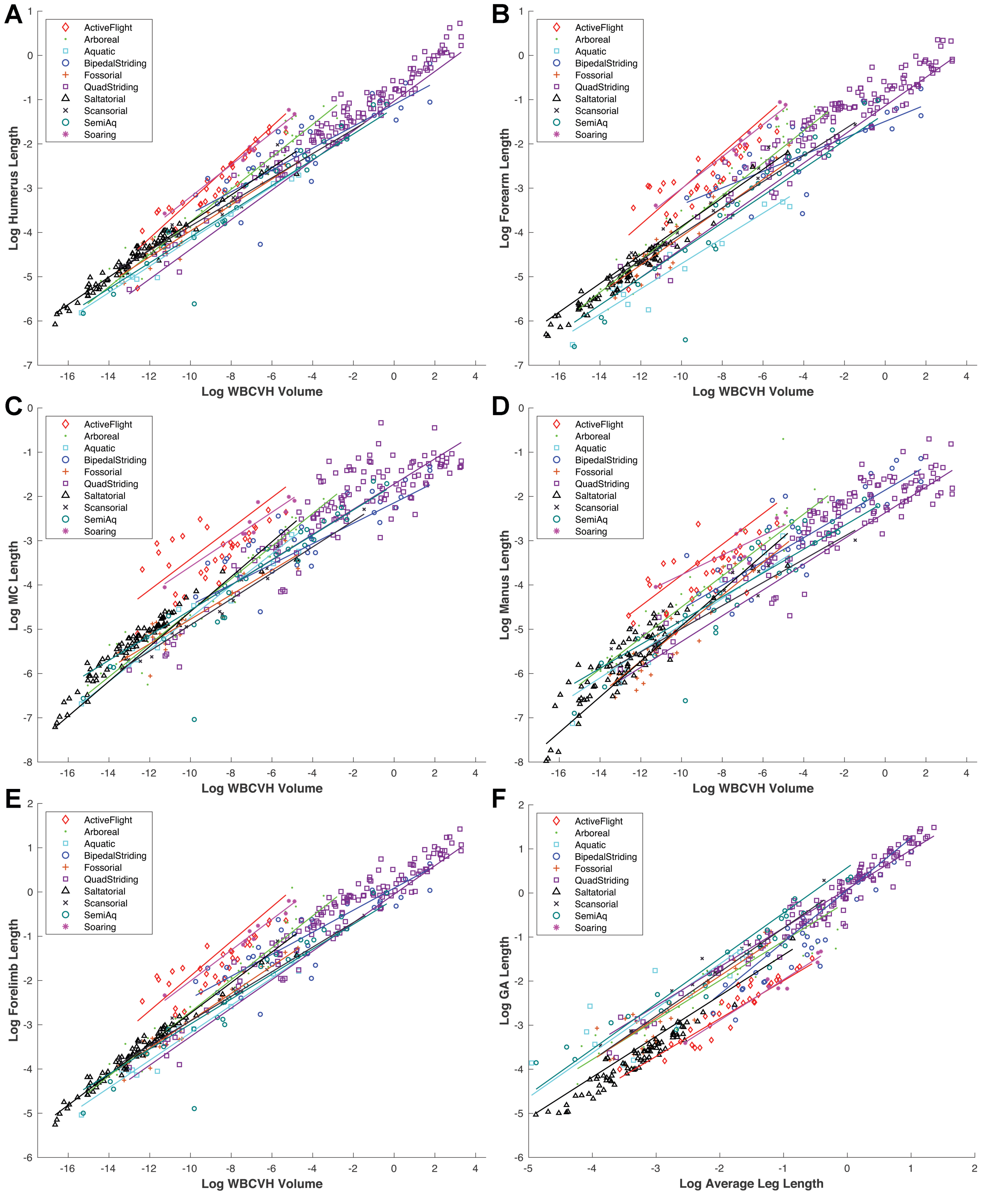

Supplementary Figure 4. Scaling in the forelimbs of different locomotor groups. Scaling relationships between forelimb body segment length versus overall body size (total whole-body skeletal convex hull volume [WBCHV]), and GA length versus average limb length in different locomotor categories, with statistical differences between phylogenetically informed linear fits tested using a phlyANCOVA. Relationships shown are (A) humerus, (B) forearm, (C) metacarpal, (D)

manus, E) total forelimb length against WBCHV, and (F) GA length against average limb length. Source data are provided as a Source Data file.

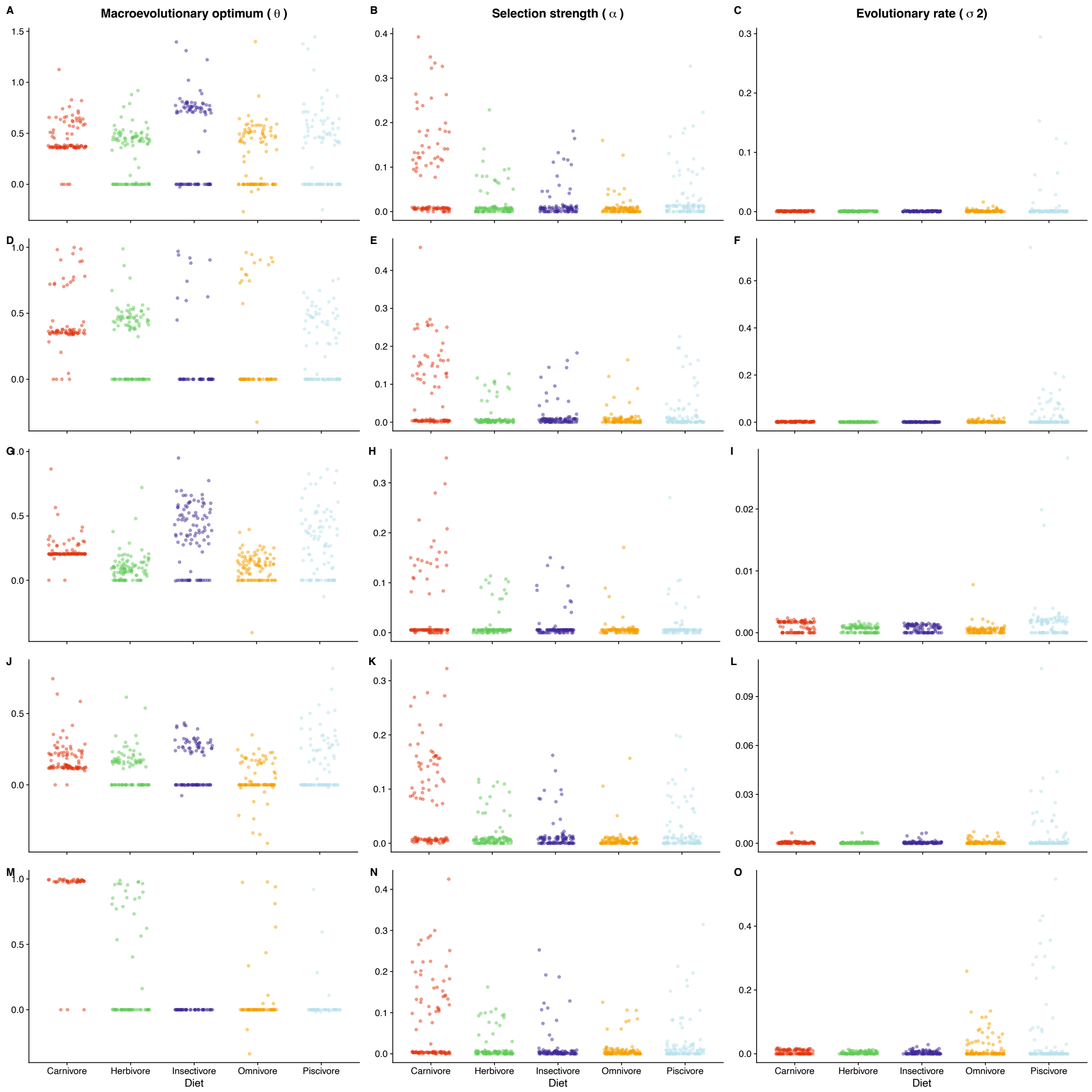

Supplementary Figure 5. Evolution of forelimb segment lengths in trophic groups. Results for the OUwie analysis for forelimb (segments and total) linear measurements. Each panel column from left to right correspond to estimates of macroevolutionary optimum, selection strength, and evolutionary rate, respectively. A-C: Humerus; D-F: Forearm; G-I: MC; J-L: Manus; M-O: Forelimb. In all panels, each point corresponds to the parameter estimate for one of the sampled simulated evolutionary regimes. Source data are provided as a Source Data file.

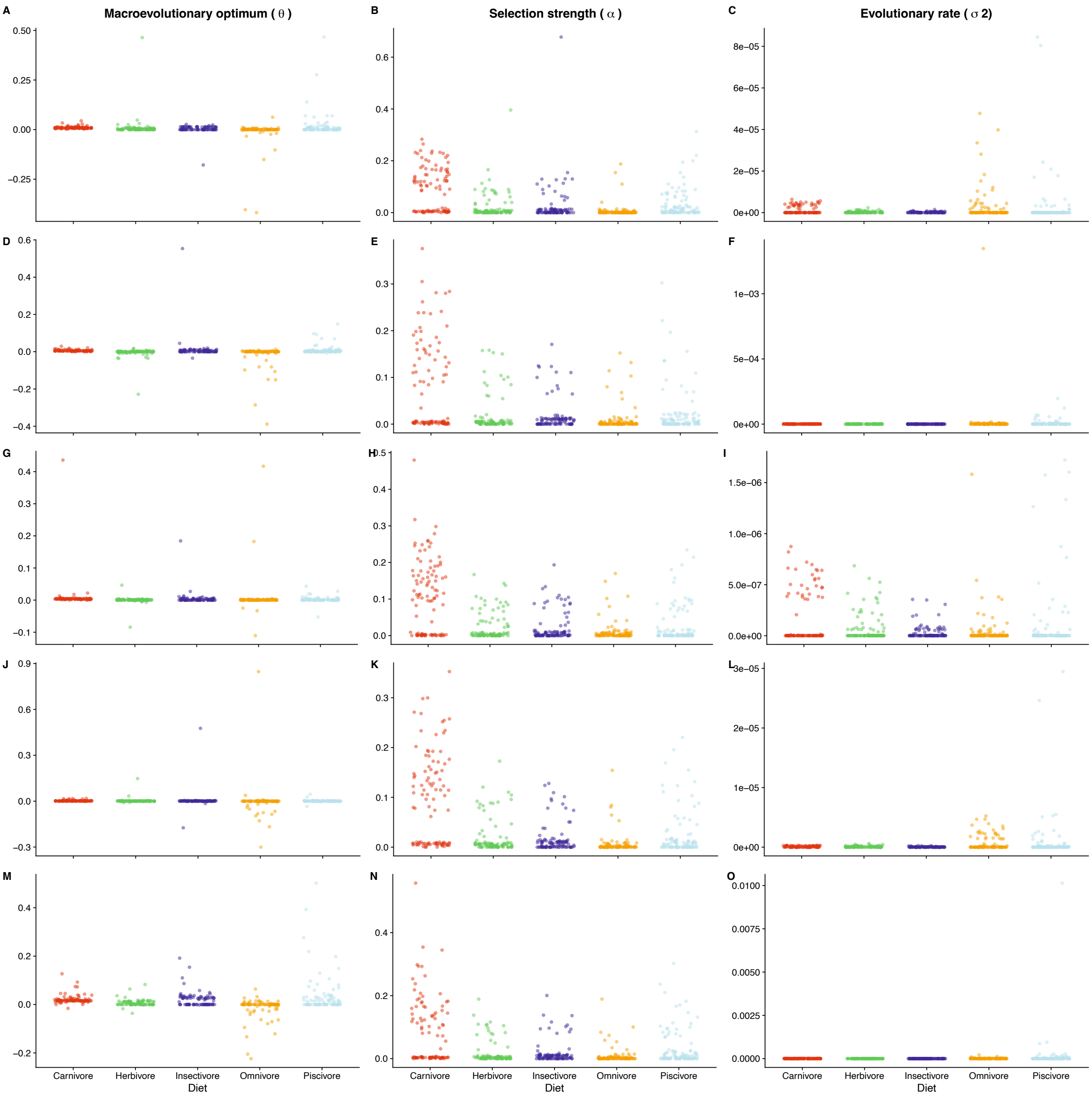

Supplementary Figure 6. Evolution of forelimb segment volumes in trophic groups. Results for the OUwie analysis for forelimb (segments and total) volume measurements. Each panel column from left to right correspond to estimates of macroevolutionary optimum, selection strength, and evolutionary rate, respectively. A-C: Humerus; D-F: Forearm; G-I: Mc; J-L: Manus; M-O: Forelimb. In all panels, each point corresponds to the parameter estimate for one of the sampled simulated evolutionary regimes. Source data are provided as a Source Data file.

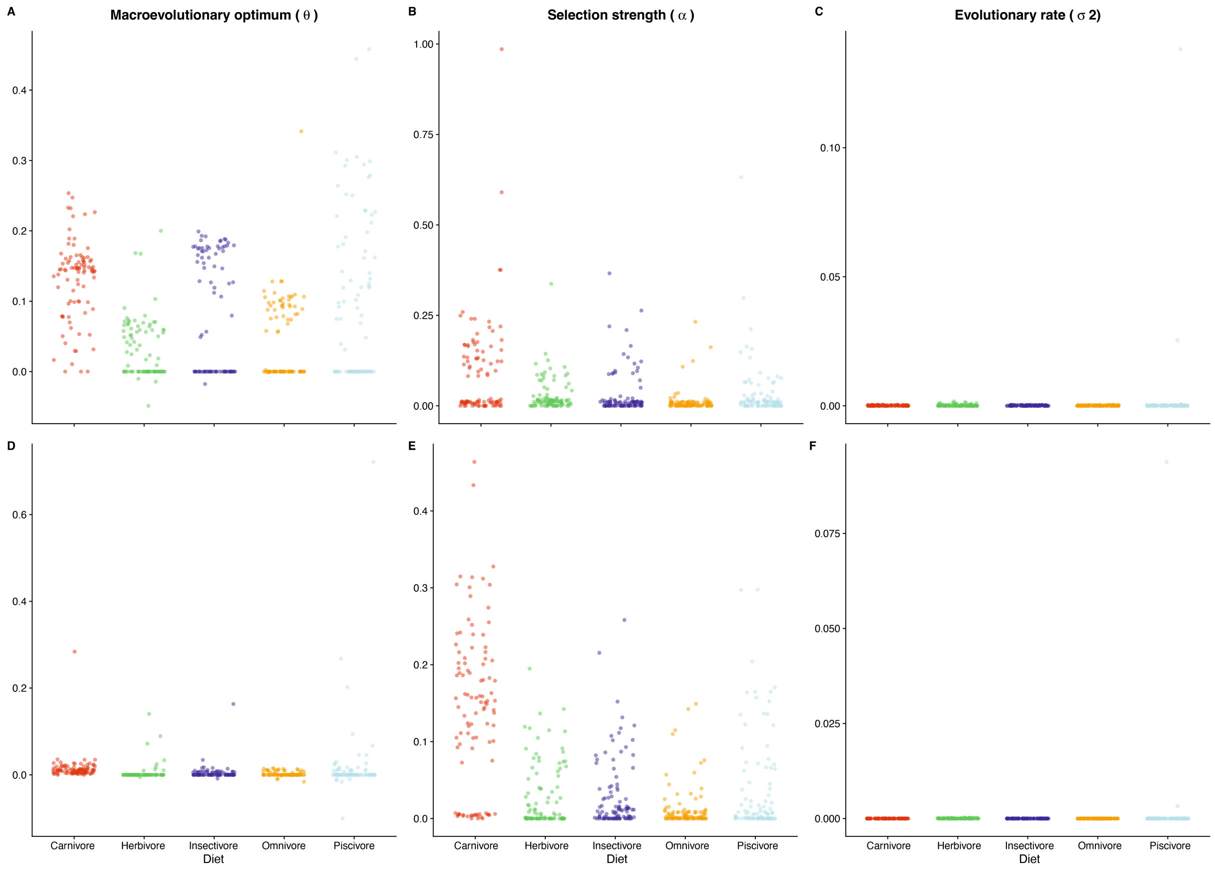

Supplementary Figure 7. Evolution of head and neck volume in trophic groups. Each panel column from left to right correspond to estimates of macroevolutionary optimum, selection strength, and evolutionary rate, respectively. A-C: Head (Skull); D-F: Neck. In all panels, each point corresponds to the parameter estimate for one of the sampled simulated evolutionary regimes. Source data are provided as a Source Data file.

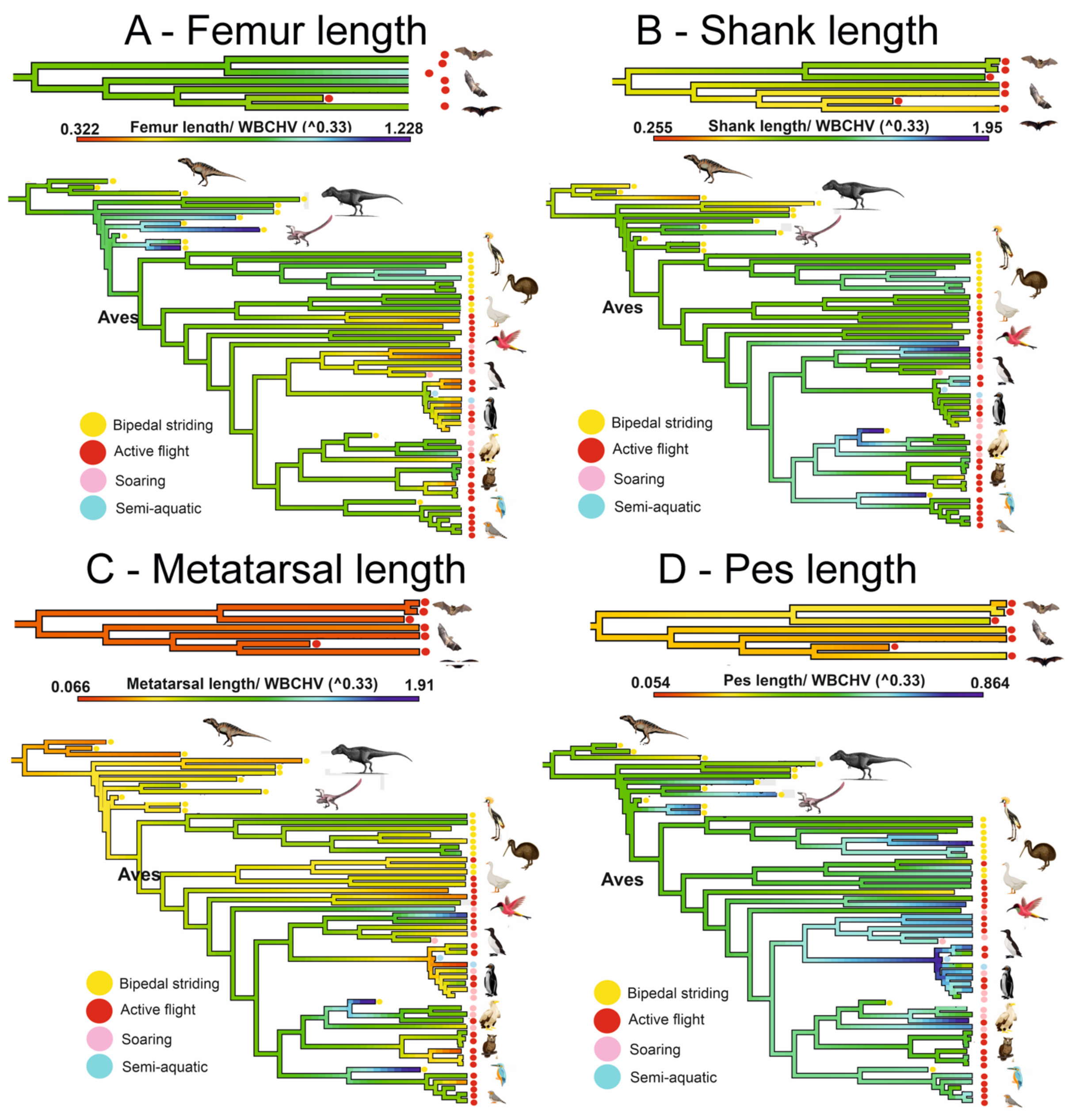

Supplementary Figure 8. Ancestral state reconstructions of hind limb segment lengths in non-avian theropod dinosaurs and birds. Colour-shaded phylogenetic trees to show the evolution of hind limb segment proportions during the evolution of locomotion in bats and across the non-avian to avian theropod transition using ancestral state reconstruction. Comparison of size normalised (A) femur, (B) shank, (C) metatarsal, and (D) pes segment lengths. WBCHV, whole-body convex hull volume. Source data are provided as a Source Data file. Animal images created with BioRender.com.

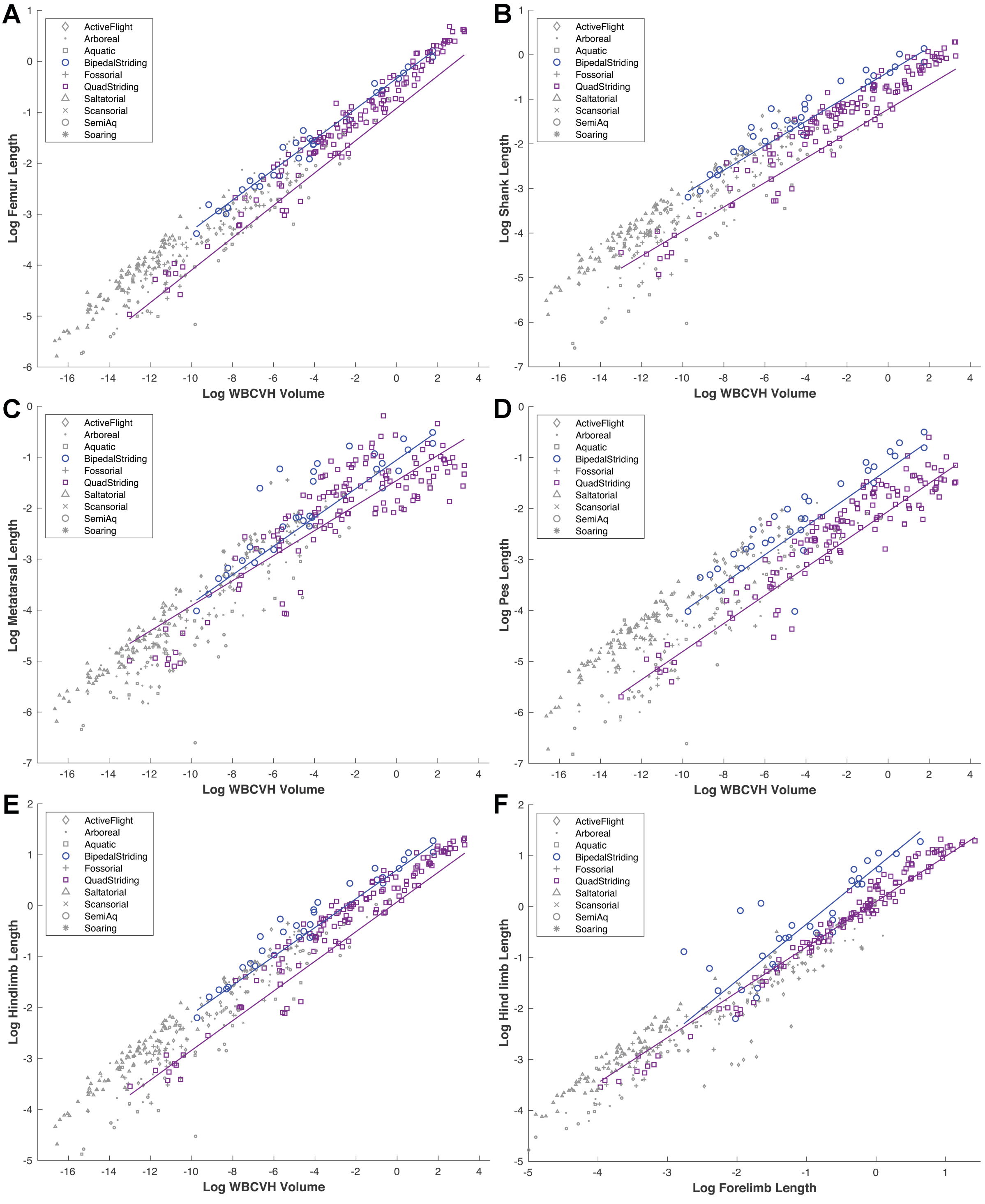

Supplementary Figure 9. Scaling of hind limb segments in different locomotor groups. Scaling relationships between hind limb body segment lengths versus overall body size (total whole-body skeletal convex hull volume [WBCHV]) in different locomotor categories, with statistical differences between phylogenetically informed linear fits tested using a phlyANCOVA. Relationships shown are (A) femur, (B) shank, (C) metatarsal, (D) pes, E)

total hind limb length and (F) hind limb against forelimb length. Source data are provided as a Source Data file.

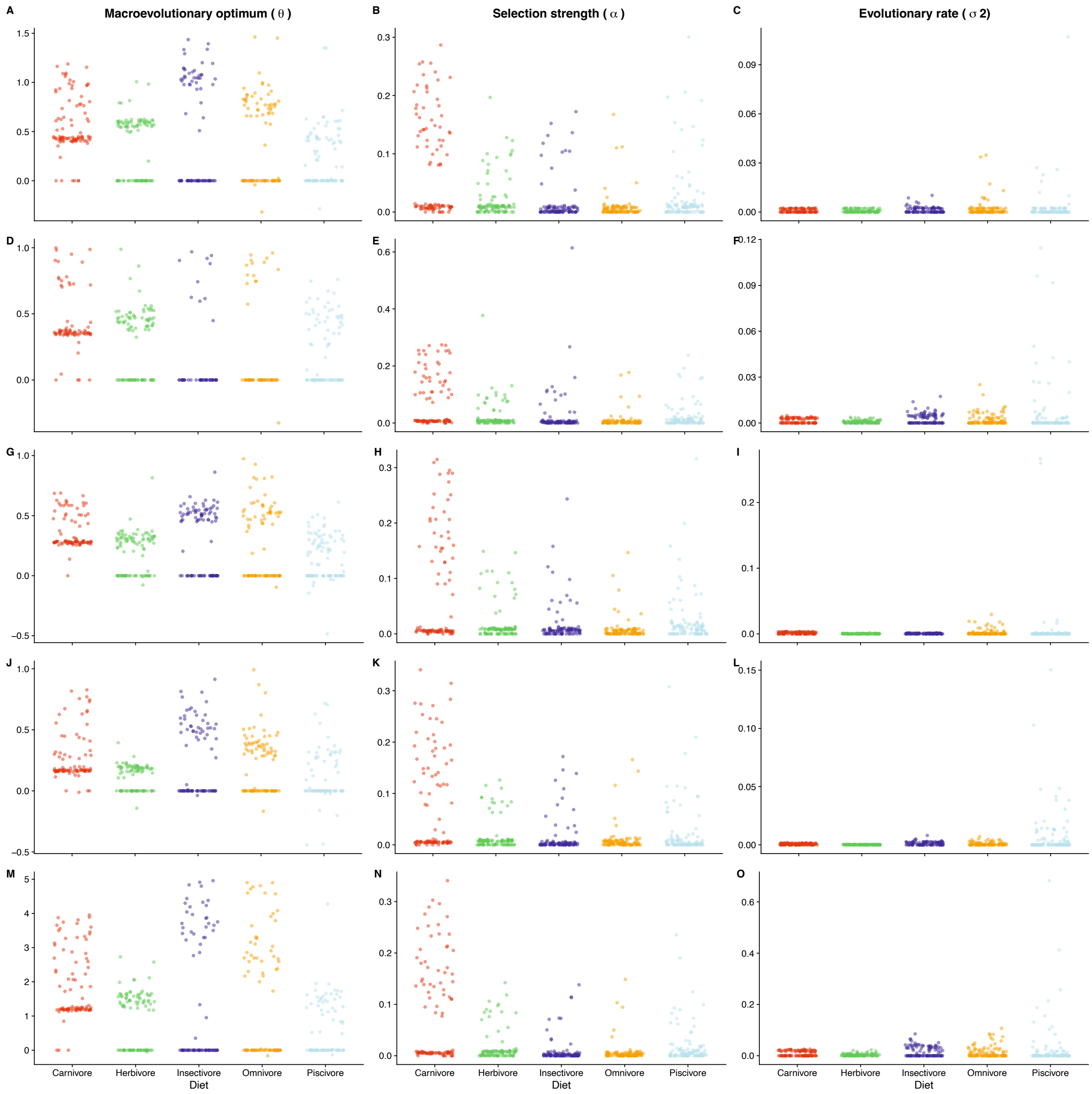

Supplementary Figure 10. Results for the OUwie analysis for hindlimb (segments and total) linear measurements. Each panel column from left to right correspond to estimates of macroevolutionary optimum, selection strength, and evolutionary rate, respectively. A-C: Femur; D-F: Shank; G-I: Metatarsal; J-L: Pes; M-O: Hindlimb. In all panels, each point corresponds to the parameter estimate for one of the sampled simulated evolutionary regimes. Source data are provided as a Source Data file.

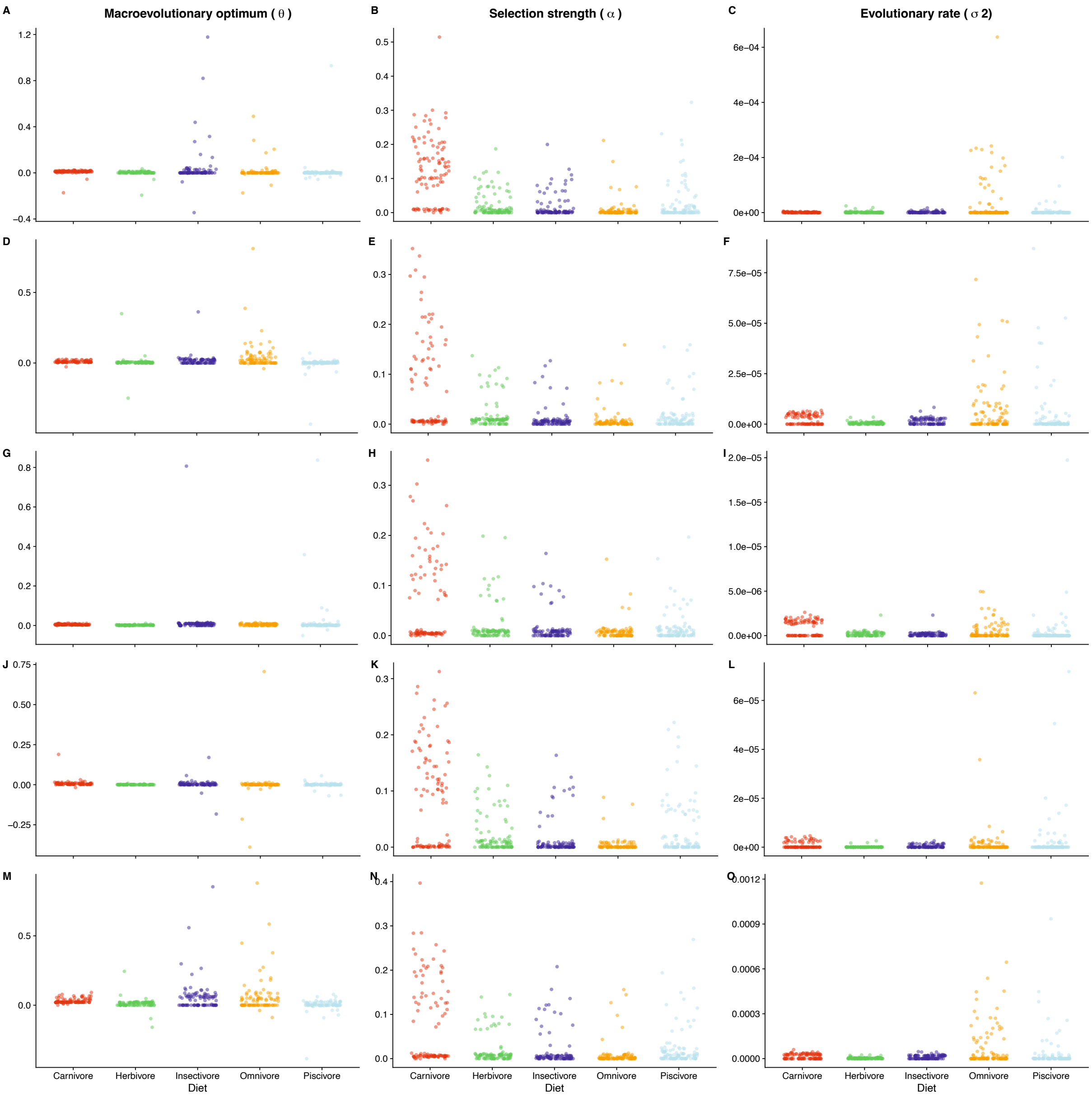

Supplementary Figure 11. Results for the OUwie analysis for hind limb (segments and total) volume measurements. Each panel column from left to right correspond to estimates of macroevolutionary optimum, selection strength, and evolutionary rate, respectively. A-C: Femur; D-F: Shank; G-I: Metatarsal; J-L: Pes; M-O: Hindlimb. In all panels, each point corresponds to the parameter estimate for one of the sampled simulated evolutionary regimes. Source data are provided as a Source Data file.

Supplementary Discussion

Hind limb evolution in theropod dinosaurs and birds. In addition to flight, the evolution of locomotion in bird-line theropods was also marked by systematic changes in the relative lengths of hind limb bones, thought to be mechanistically linked to walking and running mechanics[1-2]. Basal theropods had a long femur relative to distal limb segments (particularly the metatarsal segment) and are thought to have powered the stride from the hip[1-2]. Along the lineage to birds, the fossil record demonstrates a reversal in the relative lengths of limb bones with the femur making up a relatively small proportion of limb length and the metatarsal segment greatly enlarged[2]. This is thought to be causatively linked to a shift in joint power away from the hip in favour of the knee and ankle joints[1-2]. Our data shed light on how limb segments changed relative to overall body size during this evolutionary transition (Supplementary Figure 8A-D). Our size-normalised data suggest that femoral length did decrease in certain derived clades of non-avian theropod dinosaurs like dromaeosaurs (*Microraptor*, *Velociraptor*) and transitional taxa (*Yixianornis, Archaeopteryx*) (Supplementary Figure 8A) and remained relatively reduced in specific extant avian sub-clades (e.g., Apterygidae). However, our data also suggest that a reversal occurred in early avian evolution and that the relative size of the femur observed in basal theropods is equalled in many extant avian groups, including flying taxa (Supplementary Figure 8A). Shank length is fairly constant relative to body size across the dinosaur-bird transition and indeed within extant groups, though with noticeable shortening in certain lineages, such as Gruiformes and Cariamidae (Supplementary Figure 8B). Similar stasis is seen in metatarsal segment length relative to overall body size during theropod dinosaur evolution, but with much greater differentiation in modern groups (as noted above in the context of ecological specialisation), with many groups showing relatively short metatarsal lengths similar to basal theropod dinosaurs (Supplementary Figure 8C).

Hind limb proportions in bipedal versus quadrupedal striders. There has been significant interest in recent years in predicting bipedal versus quadrupedal locomotor habits in extinct taxa based on various aspect of limb proportions in extant taxa[3-5]. As noted in the main text, quadrupeds do not appear to exhibit particularly long forelimb lengths and in fact tend to have shorter forelimb overall than bipeds across most of their overlapping body size ranges

(Fig 6-7). However, as one might expect, bipeds do have statistically longer hind limb segments (except metatarsal length) and overall hind limb lengths than quadrupedal striders (Supplementary Figures 3, 9). Bipeds also scale with statistically lower negative allometry than quadrupeds for metatarsal length (Supplementary Figure 9C) and with positive allometry in the relationship between hind limb length versus forelimb length compared to negative allometry in quadrupedal striders (Supplementary Figure 9F; Supplementary Data 15).

Models of hind limb segment evolution in dietary categories. The trophic ecology seems to affect the evolution of the hindlimb and its segments, as all preferred models are OU models (Supplementary Data 60-61). Our results show that carnivores are subjected to stronger selection in all components and in the full hindlimb when compared to all other dietary guilds (Supplementary Figures 9-10). On the other hand, insectivores tend to evolve towards higher values of long term mean overall for both linear and volume measurements, even though the differences between guilds are not striking and vary with the evolutionary regime of the diet.

Supplementary References

1. **Allen**, V., Bates, K.T., Li, Z. and Hutchinson, J.R., 2013. Linking the evolution of body shape and locomotor biomechanics in bird-line archosaurs. *Nature*, *497*(7447), pp.104-107.
2. **Gatesy,** S.M. & Middleton, K.M. Bipedalism, flight, and the evolution of theropod locomotor diversity. *Journal of Vertebrate Paleontology* 17, 308-329. (1997).
3. **Bishop**, P.J., Bates, K.T., Allen, V.R., Henderson, D.M., Randau, M. and Hutchinson, J.R., 2020. Relationships of mass properties and body proportions to locomotor habit in terrestrial Archosauria. *Paleobiology*, *46*(4), pp.550-568.
4. **Chapelle, K. E. J.,** R. B. J. Benson, J. Stiegler, A. Otero, and J. N. Choiniere. 2020. A quantitative method for inferring locomotory shifts in amniotes during ontogeny, its application to dinosaurs, and its bearing on the evolution of posture. Palaeontology, 63, 229-242.
5. **McPhee, B.W.,** Benson, R.B.J., Botha-Brink, J., Bordy, E.M. & Choiniere, J.N. A giant dinosaur from the earliest Jurassic of South Africa and the transition to quadrupedality in early sauropodomorphs. *Current Biology,* 28, 3143-3151.
