# Supplementary material for: Body size, shape and ecology in tetrapods
Source: Nat Commun. 2022 Jul 27;13:4340. doi: 10.1038/s41467-022-32028-2 (PMC9329317; doi:10.1038/s41467-022-32028-2)
Supplement: Supplementary file 2 — Description of Additional Supplementary Files [file 41467_2022_32028_MOESM2_ESM.pdf]

File Name: Supplementary Datasets.xlsx
Description: Supplementary Data 1-89, containing summaries of statistical outputs, source information for the digital models, phylogenetic trees, age data and locomotor and dietary categorisations, as follows

Supplementary Data 1: Summary of phylogenetical generalised least squares (PGLS) information for log transformed (log10) linear parameters against body size (whole-body convex hull volume) for all taxa (N = 411). The intercept, slope, lambda, P value and upper and lower 95% confidence intervals about the slope are tabulated.

Supplementary Data 2: Summary of phylogenetical generalised least squares (PGLS) information for log transformed (log10) volume parameters against body size (whole-body convex hull volume) for all taxa (N = 411). The intercept, slope, lambda, P value and upper and lower 95% confidence intervals about the slope are tabulated.

Supplementary Data 3: Summary of results for the non-phylogenetically informed and phylogenetically informed linear and quadratic models for all taxa of linear parameters against body size (whole-body convex hull volume), SE, standard error; SEE, standard error of the estimate; AICc, Akaike information criterion; p value. Highlighted comparisons indicate the best fit model.

Supplementary Data 4: Summary of results for the non-phylogenetically informed and phylogenetically informed linear and quadratic models for all taxa of volume parameters against body size (whole-body convex hull volume) SE, standard error; SEE, standard error of the estimate; AICc, Akaike information criterion; p value. Highlighted comparisons indicate the best fit model.

Supplementary Data 5: Summary of results for the non-phylogenetically informed and phylogenetically informed linear and quadratic models for bipedal striding taxa, linear parameters against body size (whole-body convex hull volume). SE, standard error; SEE, standard error of the estimate; AICc, Akaike information criterion; p value. Highlighted comparisons indicate the best fit model.

Supplementary Data 6: Summary of results for the non-phylogenetically informed and phylogenetically informed linear and quadratic models of bipedal striding taxa, volume parameters against body size (whole-body convex hull volume). SE, standard error; SEE, standard error of the estimate; AICc, Akaike information criterion; p value. Highlighted comparisons indicate the best fit model.

Supplementary Data 7: Summary of results for the non-phylogenetically informed and phylogenetically informed linear and quadratic models for quadrupedal striding taxa, linear parameters against body size (whole-body convex hull volume). SE, standard error; SEE, standard error of the estimate; AICc, Akaike information criterion; p value. Highlighted comparisons indicate the best fit model.

Supplementary Data 8: Summary of results for the non-phylogenetically informed and phylogenetically informed linear and quadratic models for quadrupedal striding taxa, volume parameters against body size (whole-body convex hull volume). SE, standard error; SEE, standard error of the estimate; AICc, Akaike information criterion; p value. Highlighted comparisons indicate the best fit model.

Supplementary Data 9: Summary of phylogenetic generalised least squares (PGLS) information for log transformed (log10) volume and length parameters against body size (whole-body convex hull volume) for all taxa under 25kg. The intercept, slope, R^2, lambda, P value and upper and lower 95% confidence intervals about the slope are tabulated.

Supplementary Data 10: Summary of phylogenetic generalised least squares (PGLS) information for log transformed (log10) volume and length parameters against body size (whole-body convex hull volume) for all taxa over 25kg. The intercept, slope, R^2, lambda, P value and upper and lower 95% confidence intervals about the slope are tabulated.

Supplementary Data 11: Summary of phylogenetic generalised least squares (PGLS) information for log transformed (log10) volume and length parameters against body size (whole-body convex hull volume) for all taxa under 100kg. The intercept, slope, R^2, lambda, P value and upper and lower 95% confidence intervals about the slope are tabulated.

Supplementary Data 12: Summary of phylogenetic generalised least squares (PGLS) information for log transformed (log10) volume and length parameters against body size (whole-body convex hull volume) for all taxa over 100kg. The intercept, slope, R^2, lambda, P value and upper and lower 95% confidence intervals about the slope are tabulated.

Supplementary Data 13: Summary of phylogenetic generalised least squares (PGLS) information for log transformed (log10) volume and length parameters against body size (whole-body convex hull volume) for all taxa under 500kg. The intercept, slope, R^2, lambda, P value and upper and lower 95% confidence intervals about the slope are tabulated.

Supplementary Data 14: Summary of phylogenetic generalised least squares (PGLS) information for log transformed (Log10) volume and length parameters against body size (whole-body convex hull volume) for all taxa over 500kg. The intercept, slope, R^2, lambda, P value and upper and lower 95% confidence intervals about the slope are tabulated.

Supplementary Data 15: Summary of phylogenetic generalised least squares (PGLS) information for log transformed (Log) linear parameters against body size (whole-body convex hull volume) for all locomotor categories .The intercept, slope, upper and lower 95% confidence intervals about the slope, Residual standard error (RSE), lambda and p value are tabulated.

Supplementary Data 16: Summary of phylogenetic generalised least squares (PGLS) information for log transformed (Log) volume parameters against body size (whole-body convex hull volume) for all locomotor categories .The intercept, slope, upper and lower 95% confidence intervals about the slope, RSE (Residual standard error), lambda, and p value are tabulated.

Supplementary Data 17: Results from a phylogenetic ANCOVA for femur length amongst different locomotor groupings.

Supplementary Data 18: Results from a phylogenetic ANCOVA for shank length amongst different locomotor groupings.

Supplementary Data 19: Results from a phylogenetic ANCOVA for metatarsal length amongst different locomotor groupings.

Supplementary Data 20: Results from a phylogenetic ANCOVA for pes length amongst different locomotor groupings.

Supplementary Data 21: Results from a phylogenetic ANCOVA for humerus length amongst different locomotor groupings.

Supplementary Data 22: Results from a phylogenetic ANCOVA for forearm length amongst different locomotor groupings.

Supplementary Data 23: Results from a phylogenetic ANCOVA for metacarpal length amongst different locomotor groupings.

Supplementary Data 24: Results from a phylogenetic ANCOVA for manus length amongst different locomotor groupings.

Supplementary Data 25: Results from a phylogenetic ANCOVA for GA length amongst different locomotor groupings.

Supplementary Data 26: Results from a phylogenetic ANCOVA for forelimb length amongst different locomotor groupings.

Supplementary Data 27: Results from a phylogenetic ANCOVA for hindlimb length amongst different locomotor groupings.

Supplementary Data 28: Results from a phylogenetic ANCOVA for average leg length amongst different locomotor groupings.

Supplementary Data 29: Results from a phylogenetic ANCOVA for hindlimb by forelimb length amongst different locomotor groupings.

Supplementary Data 30: Results from a phylogenetic ANCOVA for GA by average leg length amongst different locomotor groupings.

Supplementary Data 31: Results from a phylogenetic ANCOVA for skull volume amongst different locomotor groupings.

Supplementary Data 32: Results from a phylogenetic ANCOVA for trunk volume amongst different locomotor groupings.

Supplementary Data 33: Results from a phylogenetic ANCOVA for femur volume amongst different locomotor groupings.

Supplementary Data 34: Results from a phylogenetic ANCOVA for shank volume amongst different locomotor groupings.

Supplementary Data 35: Results from a phylogenetic ANCOVA for metatarsal volume amongst different locomotor groupings.

Supplementary Data 36: Results from a phylogenetic ANCOVA for pes volume amongst different locomotor groupings.

Supplementary Data 37: Results from a phylogenetic ANCOVA for humerus volume amongst different locomotor groupings.

Supplementary Data 38: Results from a phylogenetic ANCOVA for forearm volume amongst different locomotor groupings.

Supplementary Data 39: Results from a phylogenetic ANCOVA for metacarpal volume amongst different locomotor groupings.

Supplementary Data 40: Results from a phylogenetic ANCOVA for manus volume amongst different locomotor groupings.

Supplementary Data 41: Results from a phylogenetic ANCOVA for neck volume amongst different locomotor groupings.

Supplementary Data 42: Results from a phylogenetic ANCOVA for forelimb volume amongst different locomotor groupings.

Supplementary Data 43: Results from a phylogenetic ANCOVA for hindlimb volume amongst different locomotor groupings.

Supplementary Data 44: Results from a phylogenetic ANCOVA for hindlimb by forelimb volume amongst different locomotor groupings.

Supplementary Data 45: Results from a phylogenetic ANCOVA for trunk by average leg length amongst different locomotor groupings.

Supplementary Data 46: Summary of phylogenetic generalised least squares (PGLS) information for log transformed (log) GA length against body size (whole-body convex hull volume) for all dietary categories. The intercept, slope, upper and lower 95% confidence intervals about the slope, R^2, lambda and P values are tabulated.

Supplementary Data 47: Summary of phylogenetic generalised least squares (PGLS) information for log transformed (log) volume parameters against body size (whole-body convex hull volume) for all dietary categories. The intercept, slope, upper and lower 95% confidence intervals about the slope, R^2, lambda and P values are tabulated.

Supplementary Data 48: Results from a phylogenetic ANCOVA for GA length amongst different dietary groupings.

Supplementary Data 49: Results from a phylogenetic ANCOVA for forelimb length amongst different dietary groupings.

Supplementary Data 50: Results from a phylogenetic ANCOVA for skull volume amongst different dietary groupings.

Supplementary Data 51: Results from a phylogenetic ANCOVA for trunk volume amongst different dietary groupings.

Supplementary Data 52: Results from a phylogenetic ANCOVA for neck volume amongst different dietary groupings.

Supplementary Data 53: Results from a phylogenetic ANCOVA for neck v skull volume amongst different dietary groupings.

Supplementary Data 54: Results from a phylogenetic ANCOVA for skull v trunk volume amongst different dietary groupings.

Supplementary Data 55: Results from a phylogenetic ANCOVA for forelimb volume against average leg length amongst different dietary groupings.

Supplementary Data 56: Body size model selection- Number of times each model was selected as the best model for Body Size (whole-body convex hull volume). OUMA: OU with multiple optima and alpha; OUMVA: OU with multiple optima, alpha and sigma.

Supplementary Data 57: Torso model selection- Number of times each model was selected as the best model for torso (trunk) (whole-body convex hull volume). OUMA: OU with multiple optima and alpha; OUMVA: OU with multiple optima, alpha and sigma.

Supplementary Data 58: Forelimb (full and segments) lengths model selection- Number of times each model was selected as the best model for forelimb (segments and total) (whole-body convex hull volume). OUMA: OU with multiple optima and alpha; OUMVA: OU with multiple optima, alpha and sigma.

Supplementary Data 59: Forelimb (full and segments) volume measurements model selection- Number of times each model was selected as the best model for forelimb (segments and total) measurements (whole-body convex hull volume). OUMA: OU with multiple optima and alpha; OUMVA: OU with multiple optima, alpha and sigma.

Supplementary Data 60: Hindlimb (full and segments) length and model selection- Number of times each model was selected as the best model for hindlimb (segments and total) (whole-body convex hull volume). OUMA: OU with multiple optima and alpha; OUMVA: OU with multiple optima, alpha and sigma.

Supplementary Data 61: Hindlimb (full and segments) volumes - Number of times each model was selected as the best model for hindlimb (segments and total) measurements (whole-body convex hull volume). OUMA: OU with multiple optima and alpha; OUMVA: OU with multiple optima, alpha and sigma.

Supplementary Data 62: Head and neck ratio measurements model selection- Number of times each model was selected as the best model for head-to-neck ratio measurements (whole-body convex hull volume). OUMA: OU with multiple optima and alpha; OUMVA: OU with multiple optima, alpha and sigma.

Supplementary Data 63: Co-efficient of variation (COV) values for all linear parameters.

Supplementary Data 64: Co-efficient of variation (COV) values for all volume parameters

Supplementary Data 65: Co-efficient of variation (COV) values for all active flight linear parameters.

Supplementary Data 66: Co-efficient of variation (COV) values for all aquatic linear parameters.

Supplementary Data 67: Co-efficient of variation (COV) values for all arboreal linear parameters.

Supplementary Data 68: Co-efficient of variation (COV) values for all bipedal striding linear parameters.

Supplementary Data 69: Co-efficient of variation (COV) values for all fossorial linear parameters.

Supplementary Data 70: Co-efficient of variation (COV) values for all quadrupedal striding linear parameters.

Supplementary Data 71: Co-efficient of variation (COV) values for all saltatorial linear parameters.

Supplementary Data 72: Co-efficient of variation (COV) values for all scansorial linear parameters.

Supplementary Data 73: Co-efficient of variation (COV) values for all semi-aquatic linear parameters.

Supplementary Data 74: Co-efficient of variation (COV) values for all soaring linear parameters.

Supplementary Data 75: Co-efficient of variation (COV) values for all active flight volume parameters.

Supplementary Data 76: Co-efficient of variation (COV) values for all aquatic volume parameters.

Supplementary Data 77: Co-efficient of variation (COV) values for all arboreal volume parameters.

Supplementary Data 78: Co-efficient of variation (COV) values for all bipedal striding volume parameters.

Supplementary Data 79: Co-efficient of variation (COV) values for all fossorial volume parameters.

Supplementary Data 80: Co-efficient of variation (COV) values for all quadrupedal striding volume parameters.

Supplementary Data 81: Co-efficient of variation (COV) values for all saltatorial volume parameters.

Supplementary Data 82: Co-efficient of variation (COV) values for all scansorial volume parameters.

Supplementary Data 83: Co-efficient of variation (COV) values for all semi-aquatic volume parameters.

Supplementary Data 84: Co-efficient of variation (COV) values for all soaring volume parameters.

Supplementary Data 85: List of taxa included and sources of the 3D skeletal models.

Supplementary Data 86: List of the phylogenetic trees merged to construct the tree used in this study, including their source information.

Supplementary Data 87: First and last occurrence dates used in the time-calibrated tree in this study, including source information.

Supplementary Data 88: Literature and sources used to assign studied species to locomotor categories.

Supplementary Data 89: Literature and sources used to assign studied species to dietary categories.

File Name: Supplementary Code 1
Description: Example code to run pGLS and quadratric fits for on all taxa segment size vs body size regression.

File Name: Supplementary Code 2
Description: Example code for pGLS and phylANCOVA in dietary categories.

File Name: Supplementary Code 3
Description: Example code for pGLS and phylANCOVA in locomotor categories.

File Name: Supplementary Code 4
Description: Code required to repeat our Stochastic Character Mapping and models of continuous trait evolution in R.
