# Supplementary material for: Body size, shape and ecology in tetrapods
Source: Nat Commun. 2022 Jul 27;13:4340. doi: 10.1038/s41467-022-32028-2 (PMC9329317; doi:10.1038/s41467-022-32028-2)
Supplement: Supplementary file 4 — Supplementary Code 1-4 [file 41467_2022_32028_MOESM4_ESM.zip › SupplementaryCode/SupplementaryCode_README.rtf]

Body size, shape and ecology in tetrapodsMaher et al. Supplementary Code for statistical analyses in RThe directory “~\SupplementaryCode/PGLS_PHYLANCOVA/ contains example code and input data for pGLS and phylANCOVA analyses, as follows:SupplementaryCode1.txt = pGLS and quadratic fit exampleSupplementaryCode2.txt = pGLS & PhylANCOVA on trophic categoriesSupplementaryCode3.txt = pGLS & PhylANCOVA on locomotor categoriesAliceLinear.csv = linear input dataAliceVolume.csv = volume input datastraptreenew.nex = tree fileThe directory “~\SupplementaryCode/OuwieAnalysis/ contains example code (within the directory ‘analyses’) and input data (within the directory ‘’data’) for the Ouwie trait evolution analyses. 
